# Ml proteins from *Mesorhizobium loti* and MucR from *Brucella abortus*: an AT-rich core DNA-target site and oligomerization ability

**DOI:** 10.1038/s41598-017-16127-5

**Published:** 2017-11-17

**Authors:** Ilaria Baglivo, Luciano Pirone, Emilia Maria Pedone, Joshua Edison Pitzer, Lidia Muscariello, Maria Michela Marino, Gaetano Malgieri, Andrea Freschi, Angela Chambery, Roy-Martin Roop II, Paolo Vincenzo Pedone

**Affiliations:** 1 Department of Environmental, Biological and Pharmaceutical Sciences and Technologies, University of Campania “Luigi Vanvitelli”, Caserta, 81100 Italy; 2 Institute of Biostructures and Bioimaging, C.N.R., Naples, 80134 Italy; 3 Department of Microbiology and Immunology, Brody School of Medicine, East Carolina University, Greenville, NC USA

## Abstract

*Mesorhizobium loti* contains ten genes coding for proteins sharing high amino acid sequence identity with members of the Ros/MucR transcription factor family. Five of these Ros/MucR family members from *Mesorhizobium loti* (Ml proteins) have been recently structurally and functionally characterized demonstrating that Ml proteins are DNA-binding proteins. However, the DNA-binding studies were performed using the Ros DNA-binding site with the Ml proteins. Currently, there is no evidence as to when the Ml proteins are expressed during the *Mesorhizobium lo* *ti* life cycle as well as no information concerning their natural DNA-binding site. In this study, we examine the *ml* genes expression profile in *Mesorhizobium loti* and show that *ml1*, *ml2*, *ml3* and *ml5* are expressed during planktonic growth and in biofilms. DNA-binding experiments show that the Ml proteins studied bind a conserved AT-rich site in the promoter region of the *exoY* gene from *Mesorhizobium loti* and that the proteins make important contacts with the minor groove of DNA. Moreover, we demonstrate that the Ml proteins studied form higher-order oligomers through their N-terminal region and that the same AT-rich site is recognized by MucR from *Brucella abortus* using a similar mechanism involving contacts with the minor groove of DNA and oligomerization.

## Introduction

The Ros/MucR transcription factor family^1–3^ includes proteins such as Ros from *Agrobacterium tumefaciens* ^4^ and MucR from *Brucella abortus* ^2^ responsible for the expression regulation of virulence genes and MucR from *Sinorhizobium meliloti* involved in the gene expression regulation necessary for the symbiosis process established by *Rhizobia* with plant^5^. Furthermore MucR from *Caulobacter crescentus* has been described being involved in cell cycle regulation^6^. The Ros protein from *Agrobacterium tumefaciens* was the first prokaryotic zinc-finger DNA-binding protein to be identified and its DNA-binding domain has been well characterized both structurally and functionally^7–12^. In *Mesorhizobium loti*, a nitrogen fixing bacterium that establishes symbiosis with *Lotus japonicus* by forming root nodules, ten Ros/MucR homologues are present and five of them (Ml1-Ml5) have been shown to have the capacity to function as DNA-binding proteins like Ros^1^. To function as DNA-binding proteins, the DNA-binding domains of Ml1, Ml2 and Ml3 require a zinc atom to fold like Ros, whereas the DNA-binding domains of Ml4 and Ml5 are able to fold and bind DNA in the absence of the metal ion^1,9,13^. The Ml4 and Ml5 are able to fold in the absence of zinc due to the presence of a more complex network of hydrogen bonds and a more extensive hydrophobic core than what is found in the zinc-binding domains Ml1-Ml3 and the Ros protein^10^. Interestingly, the DNA-binding domains of Ml1, Ml2 and Ml3 show a heterogeneous zinc-coordination sphere that includes an aspartic acid as the second coordinating residue instead of the cysteine present in the Ros zinc-finger domain^1,12,14^. Despite having different zinc-coordinating residues, the Ml proteins share more than 40% sequence identity with the Ros protein from *Agrobacterium tumefaciens* ^1^.The high amino acid sequence homology between Ros and the Ml proteins is consistent with the fact that the Ml proteins have the capacity to bind to the AT-rich Vir Box^15^, the natural target site for the Ros protein^1^. In spite of these detailed structural and functional studies conducted with the Ml proteins and the Vir Box, the native DNA-target sites for the Ml proteins have not been defined and the functional role of the N-terminal region of the Ml proteins has not been determined. In addition, there is no information as to when the *ml* genes are expressed during the *Mesorhizobium loti* life cycle.

Only a few studies have been conducted so far to investigate the nature of the DNA-target sites for the Ros/MucR family of proteins and these studies have mostly indicated that these proteins bind to long degenerated AT-rich sequences, but without providing detailed mechanistic information^6^. Many other prokaryotic DNA-binding proteins recognize similar AT-rich DNA sequences and their binding often involves contacts between the protein and the minor groove of the DNA^16^. Examples of prokaryotic proteins that bind AT-rich DNA sequences include the TATA-box Binding Proteins, IHF (Integration Host Factor) as well as the histone like proteins of the H-NS family^16,17^. Interestingly, members of H-NS protein family from the alpha, beta and gamma proteobacteria^18^ bind DNA in a distinct manner by recognizing specific structural features in the minor groove of AT-rich DNA sequences^17,18^.

In this study, we determine for the first time the expression profiles of the genes encoding the Ml1-Ml5 proteins from *Mesorhizobium loti* ^1^ during growth under both planktonic condition and in biofilms. We also define the natural DNA-target sites recognized by the Ml proteins and by the MucR protein from *Brucella abortus*. Our DNA-binding studies demonstrate that an AT-rich sequence containing a T-A step is the “core” DNA-binding site for the Ml proteins and the MucR protein from *Brucella abortus*. In addition, we examined the oligomerization properties of the Ml proteins and MucR from *Brucella abortus* and show that the N-terminal region of the proteins is responsible for oligomer formation.

## Results

### *ml1, ml2, ml3*, and *ml5* genes are expressed in *Mesorhizobium loti* both during planktonic growth and in biofilms

**Figure 1:**
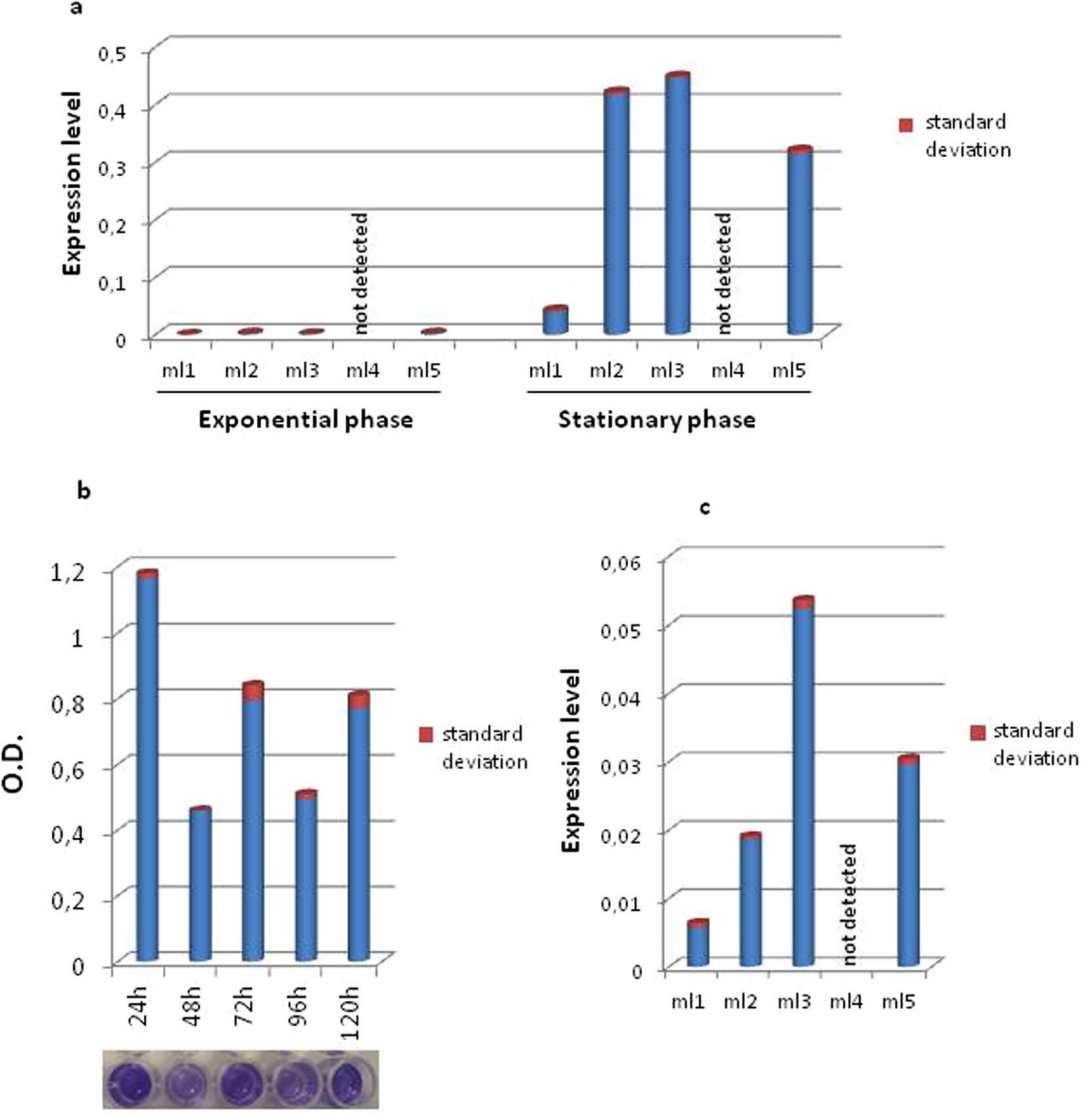
Expression profile of *ml* genes and biofilms formation. (**a**) *ml* genes expression level during exponential and planktonic growth; (**b**) graphic representation of biofilms attachment and detachment phases; (**c**) *ml* genes expression level in biofilms at 24 h. For all the RT-qPCR values t-test, P < 0.05.

To evaluate the expression profiles of the genes encoding the five Ml proteins in *M*. *loti* during planktonic growth, RNA was isolated from bacterial cells collected during both the exponential phase and stationary phase for analysis by RT-qPCR. The results obtained indicated that the *ml1*, *ml2*, *ml3* and *ml5* genes are all expressed during stationary phase with the *ml1* displaying considerably lower expression than the other three genes, but all four are barely expressed in the exponential growth phase (Fig. 1 a). In contrast, no expressions of the *ml4* transcript was detected under either of the two conditions tested. Given that two species of Mesorhizobia, *Mesorhizobium huakuii* and *Mesorhizobium tianshanense* have been shown to form biofilms^19^, we also examined the ability of *M*. *loti* to form biofilms and found that *M*. *loti* does have the capacity to form biofilms with an attachment and detachment phase every 24 h (Fig. 1 b). Based on these results, the expression of the *ml* genes was further investigated in biofilms by RT-qPCR and the expression patterns appear to be similar to those found in the stationary phase of planktonic growth, with *ml1* displaying considerably lower expression than *ml2*, *ml3* and *ml5* as well as no expression of *ml*4 (Fig. 1 c). However, the overall expression levels of the *ml1*, *2*, *3* and *5* genes are lower in biofilms than they are in the planktonic stationary phase.

### The core DNA-target site of Ml1 and Ml2 is a five base pair AT-rich sequence containing a T-A step

**Figure 2:**
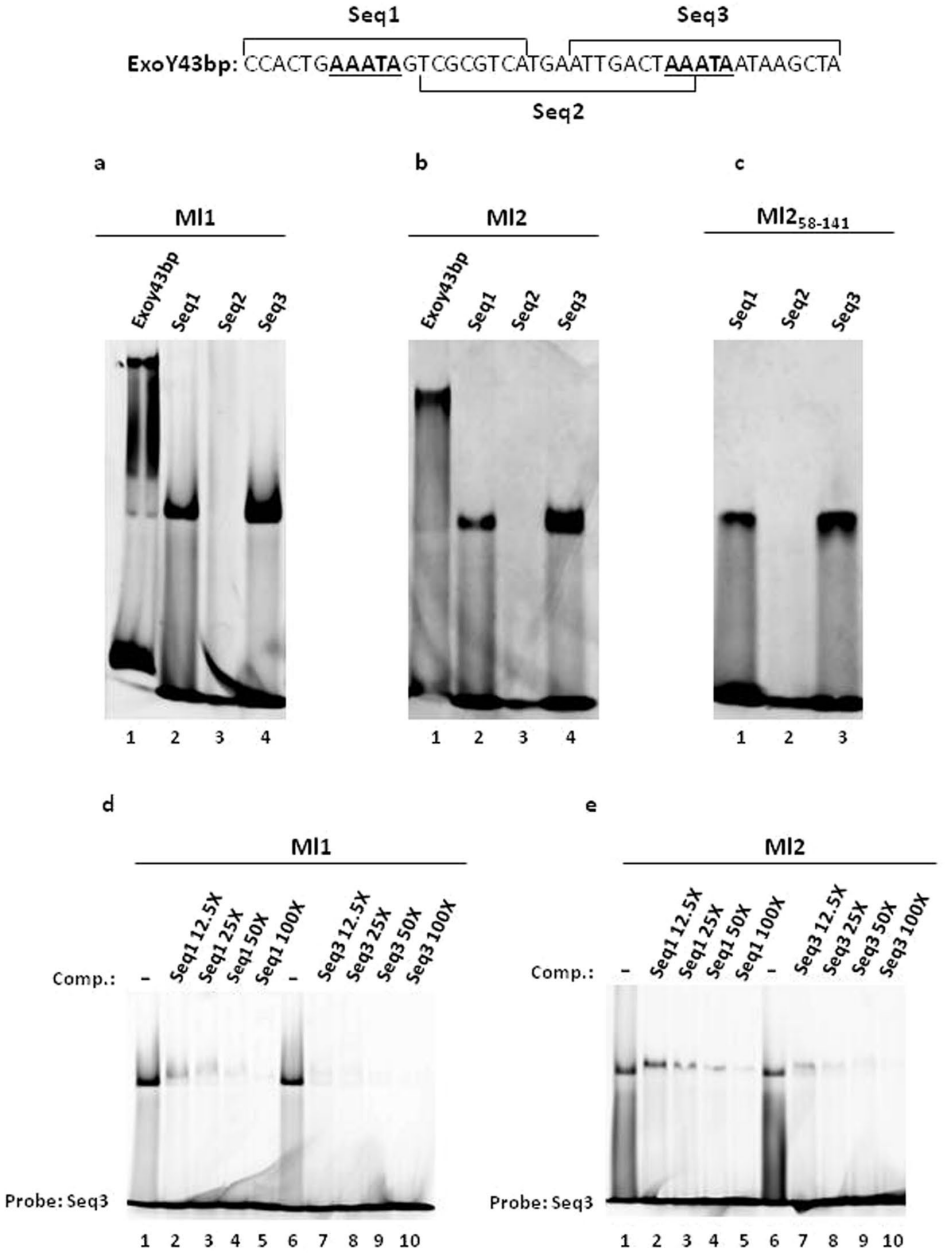
Mls binding to the Exo43bp, Seq1, Seq2 and Seq3 double-stranded oligonucleotides by EMSA. The nucleotide sequence of the Exo43bp is shown and the sequences of the three 20 bp long oligonucleotides, Seq1, Seq2 and Seq3, are also indicated. (**a**) EMSA with Ml1; (**b**) EMSA with Ml2; (**c**) EMSA with Ml2_58–141_; (**d**) competition assays with Ml1 using Seq1 and Seq3 as competitors (Comp.) and the FAM-labelled Seq3 as a probe; (**e**) competition assays with Ml2 using Seq1 and Seq3 as competitors (Comp.) and the FAM-labelled Seq3 as a probe. The amounts of the competitors are indicated on the top of the lanes as excess with respect to the probe (from 12.5-fold to 100-fold excess). Full-length gels are presented in Supplementary Figs S8–S12.

In an attempt to identify the native DNA-target site, we aligned the entire *M*. *loti* genome^20^ with the Vir Box sequence used in previous studies demonstrating that the Ml proteins functioned as DNA-binding proteins^1^. Based on this anlaysis, we identified a putative DNA target sequence (Exoy43bp) for the Ml proteins positioned −82 bp from the ATG start codon in the promoter region of the *M*. *loti exoY* gene (Supplementary Fig. S1). In *S*. *meliloti*, the *exoY* gene encodes a galactosyl transferase that carries out the first step of succinoglycan biosynthesis^21,22^ and bioinformatic analyses indicate that *S*. *meliloti* and *M*. *loti* share the same *exo* genes^23^. The Vir Box and Exoy43bp share 51% sequence identity, and the latter sequence has a base pair composition of 65% AT and 35% GC. The Ml1, Ml2, Ml3 and Ml5 share 45% amino acid identity with each other (Supplementary Fig. S2) and we chose to investigate the DNA-binding ability of Ml1 and Ml2 (Mls) to the Exoy43bp putative target site using an Electrophoretic Mobility Shift Assay (EMSA). The results obtained show that the Mls bind the Exoy43bp sequence, but they form more than one set of bands in the EMSA experiments (Fig. 2, a, lane 1, and b, lane 1). The presence of multiple bands suggests that more than one target site is present in the sequence and that more than one molecule of the protein is binding to the Exoy43bp DNA. To identify the region(s) of the Exoy43bp sequence that are bound by the Mls, we tested the ability of Ml1 and Ml2 to recognize three partially overlapping 20 bp long oligonucleotides (Seq1, Seq2, Seq3) derived from Exoy43bp (Fig. 2). The results show that both Ml1 and Ml2 bind to either Seq1 or Seq3, but they fail to recognize Seq2 (Fig. 2, a, lanes 2–4 and b, lanes 2–4) even when a four-fold molar excess of protein was used (Supplementary Fig. S3). To further test these three overlapping sequences, we also tested their binding to just the C-terminal DNA-binding domain of Ml2 and we obtained the same results as with the entire Ml2 (Fig. 2 c, lanes 1–3). In addition, competition assays demonstrate that the Mls bind Seq3 with higher affinity than Seq1 (Fig. 2 d and e).

**Figure 3:**
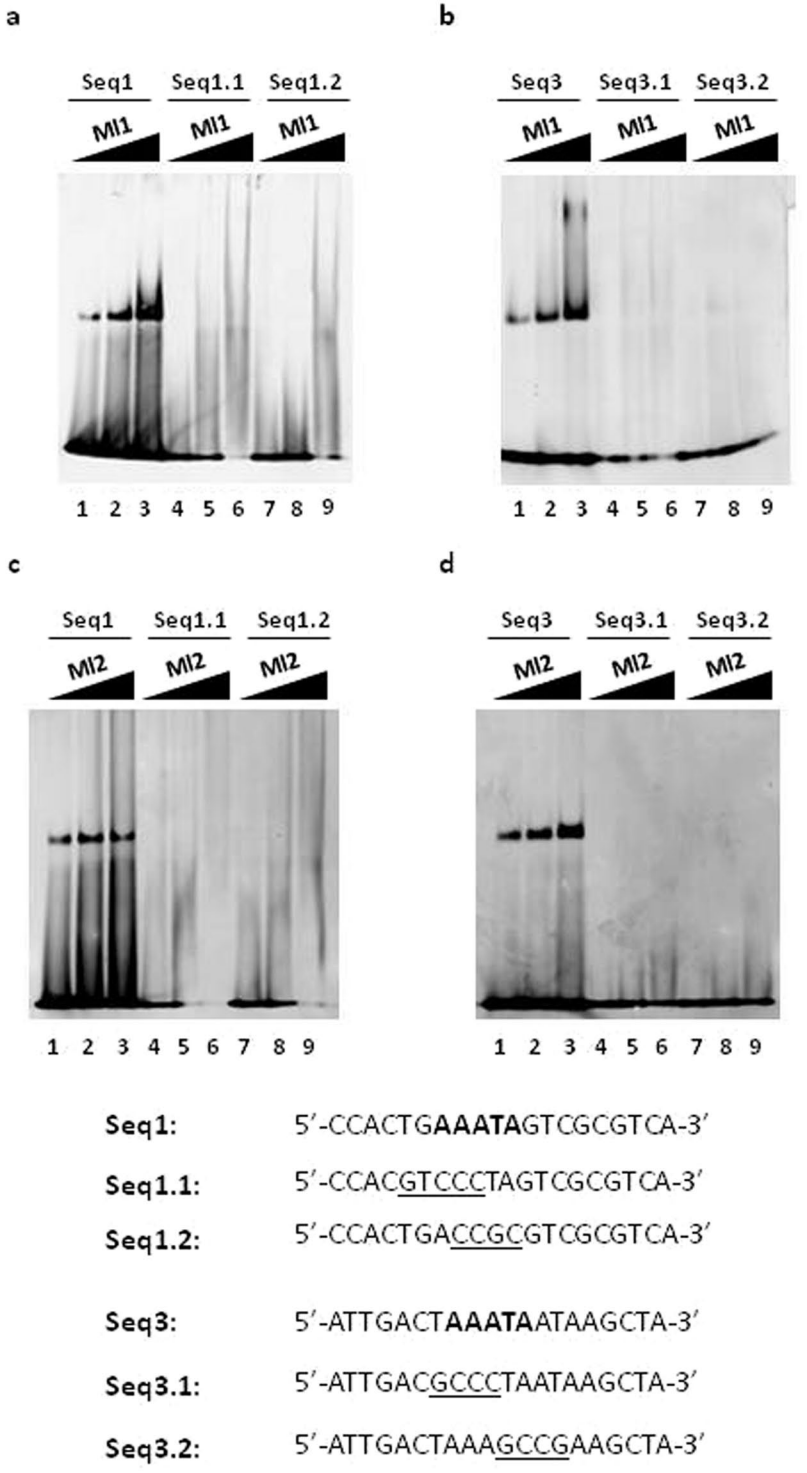
Mls binding to the Seq1 and Seq3 mutant double-stranded oligonucleotides. The sequences of the oligonucleotides tested are shown. The five base pairs shared by Seq1 and Seq3 are in bold and the mutated bases in Seq1.1, Seq1.2, Seq3.1 and Seq3.2 are underlined. Three different quantities of each protein (0.5 μg, 1 μg, and 2 μg) were used with each double-stranded oligonucleotides. The growing quantities of protein used is indicated on the top of each lane (**a**) EMSA of Ml1 with Seq1, Seq1.1 and Seq1.2; (**b**) EMSA of Ml1 with Seq3, Seq3.1, Seq3.2; (**c**) EMSA of Ml2 with Seq1, Seq1.1 and Seq1.2; (**d**) EMSA of Ml2 with Seq3, Seq3.1, Seq3.2. Full-length gels are presented in Supplementary Figs S13–S16.

Seq1 is 50% AT-rich whereas Seq3 is 75% AT-rich and this suggests that the higher AT richness plays an important role in the higher binding affinity observed with Seq3. In addition, we found that Seq1 and Seq3, but not Seq2, contain the sequence 5′-AAATA-3′ followed by either a guanine in the case of Seq1 (5′-AAATAG-3′) or by an adenine in the case of Seq3 (5′-AAATAA-3′). Based on these two observations, we hypothesized that the Mls target site was centered on the five base pair sequence 5′-AAATA-3′ in Seq1 and Seq3 and this explained why we saw more than one band in the EMSA experiments with the full Exoy43bp oligonucleotide. To test this hypothesis, we prepared two mutant versions of Seq1 (Seq1.1 and Seq1.2) and two mutant versions of Seq3 (Seq3.1 and Seq3.2) and in all mutants the 5′-AAATAG/A-3′ hexamer was disrupted (Fig. 3). These sequences were tested in EMSA studies with Ml1 and Ml2 and the results show that under the condition tested, the Mls are not able to form a stable complex with any of the mutated versions of either Seq1 (Fig. 3, a, lanes 4–9, c, lanes 4–9) or Seq3 (Fig. 3, b, lanes 4–9, d, lanes 4–9) even at a four-fold molar excess of protein. Altogether, these results with the mutated versions of Seq1 and Seq3 support the notion that the DNA-target site for the Mls present in Seq1 and Seq3 are centered on the sequence 5′-AAATA-3′.

**Figure 4:**
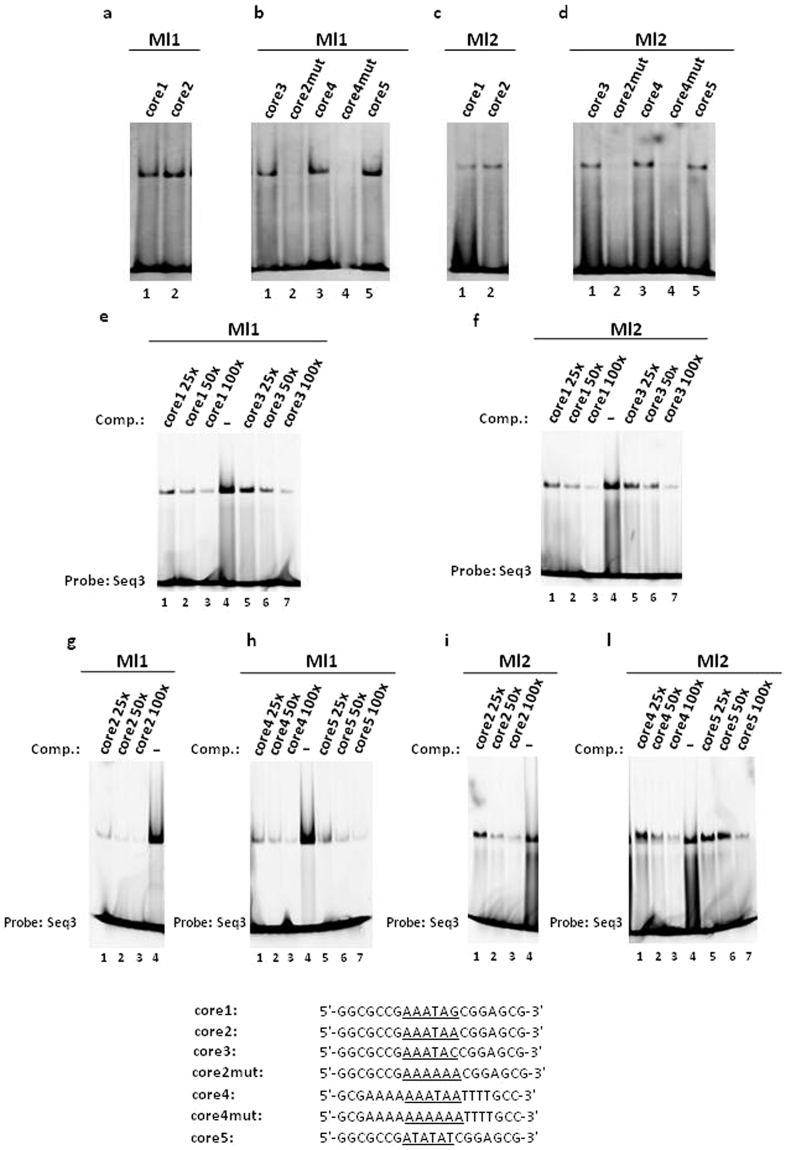
Mls binding to the core double-stranded oligonucleotides (**a**–**d**) and competition experiments (**e–l**). The sequences of the oligonucelotides tested as target sites and as competitors (Comp.) are shown. The competition experiments were perfomed by adding 25-, 50- or 100-fold excess of each competitor to the reaction mixture with respect to the amount of the FAM-labelled Seq3 double-stranded oligonucleotide used as the probe. Full-length gel are presented in Supplementary Figs S17–S26.

As mentioned above, Seq1 has a higher GC content than Seq3 and this includes the base pair immediately following the proposed 5′-AAATA-3′ target site. To investigate whether or not this base pair has an influence on higher binding affinity observed with Seq3, two 20 bp oligonulceotides were designed. One sequence (core1) contained the 5′-AAATAG-3′ hexamer and the other (core2) contained the 5′-AAATAA-3′ hexamer positioned at the center of a scrambled sequence (Fig. 4). In the EMSA experiments, the Mls are able to bind to both the core1 and core2 sequences as expected (Fig. 4, a, lanes 1, 2, and c, lanes 1, 2) and competition experiments demonstrate that the two oligonucleotides bind with similar affinities to the Mls (Fig. 4, e, lanes 1–4, g, lanes 1–4, f, lanes 1–4 and i, lanes 1–4). To further test the role of this position, we designed a third oligonucleotide (core3) in which the sequence 5′-AAATA-3′ is followed by a cytosine to investigate whether the presence of a pyrimidine could affect DNA-binding. The results obtained by EMSAs (Fig. 4, b, lane 1 and d, lane 1) and competition assays (Fig. 4, e, lanes 4–7 and f, lanes 4–7) show that the core3 binds with similar affinity to the Mls in comparison to the core1 and core2 sequences and this demonstrates that the presence of a pyrimidine following the sequence 5′-AAATA-3′ does not affect the DNA-binding. The latter result, together with the result obtained using the core1 containing the hexamer 5′- AAATAG-3′, demonstrates that an AT-rich sequence of five base pairs is necessary and sufficient for DNA-binding to the Mls. Furthermore, all the core oligonulceotides bound by the Mls share a central T-A step. This T-A step is responsible for DNA shape readout recognition^16^ because of its flexibility, which makes the minor groove wider than it is in stiffer A-tracts that narrow the minor groove^16,24^.

**Figure 5:**
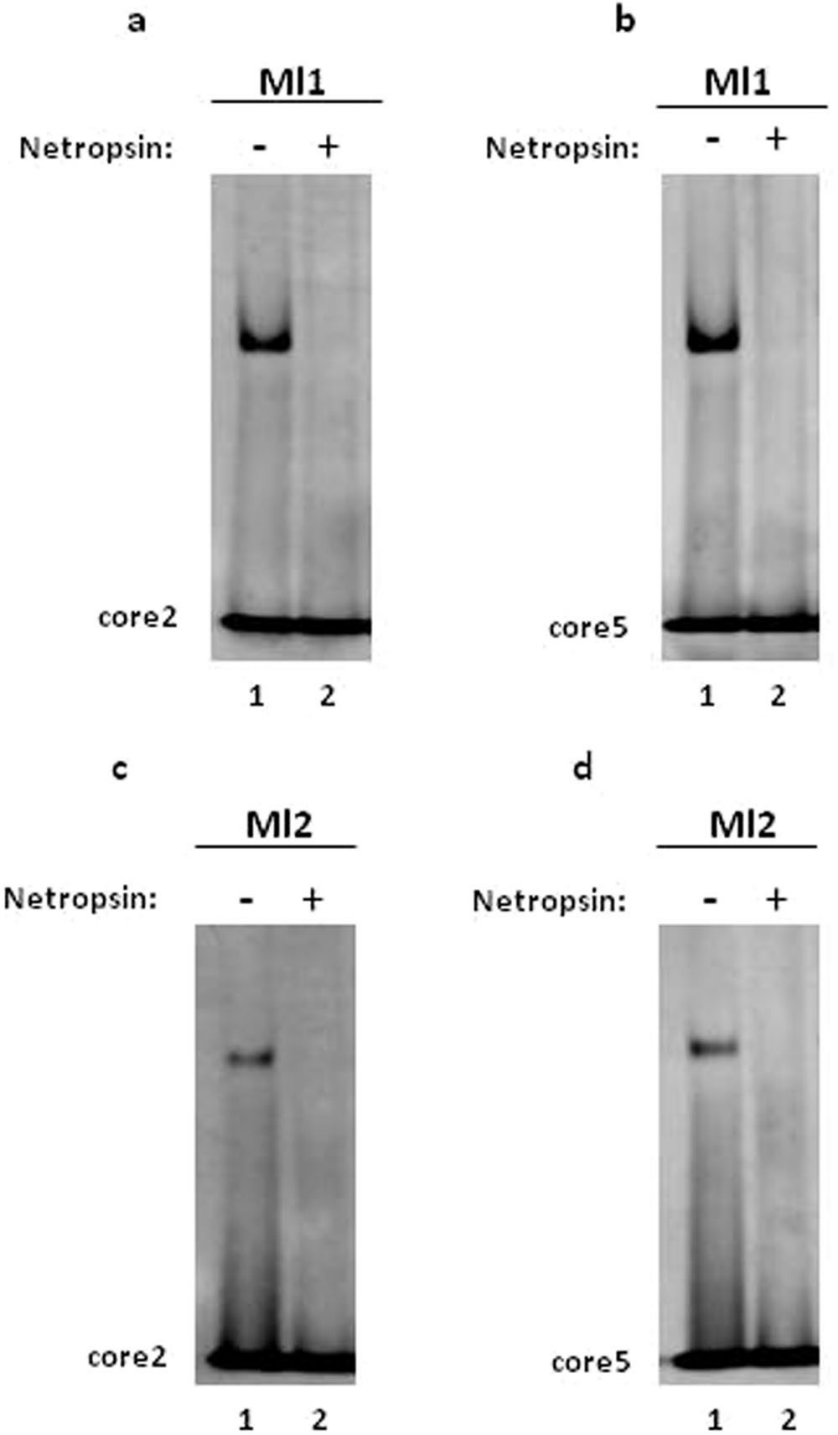
Competition assays of Mls DNA-binding with netropsin. (**a**) Competition assay with Ml1 binding to double-stranded core2 and (**b**) to double-stranded core5 in the presence of a 1:1 molar ratio of netropsin; (**c**) competition assay of Ml2 binding to double-stranded core2 and (**d**) to double-stranded core5 in the presence of a 1:1 molar ratio of netropsin. Full-length gels are presented in Supplementary Figs S27–S30.

The presence of a T-A step at the center of DNA-target sequence is known to play a pivotal role in the minor groove recognition of DNA by proteins such as H-NS^17,25^. To test the hypothesis that the T-A step has a pivotal role in DNA-binding of Mls, we prepared a mutant of the core2 oligonucleotide (core2mut) in which the thymine of the hexamer 5′-AAATAA-3′ is changed to adenine (Fig. 4). Under the condition tested in the EMSA experiments, the Mls failed to bind to the core2mut (Fig. 4, b, lane 2 and d, lane 2) even when a four-fold molar excess of protein was used (Supplementary Fig. S4). This results suggest that the central T-A step has a pivotal role for DNA-binding by the Mls. Furthermore, we investigated whether the DNA-binding affinity of Mls could be altered by extending the target sequence with the addition of A-tracts defined as four or more succeeding adenines^16^. To test this, an oligonucleotide (core4) was prepared, which contains the 5′-AAATAA-3′ hexamer that is extended on the 5′ end by four additional adenines and on the 3′ end by four additional thymines (Fig. 4). The core4 sequence was tested in EMSA experiments as a target site for Mls (Fig. 4, b, lane 3 and d, lane 3) as well as in competition assays (Fig. 5, h, lanes 1–4 and l, lanes 1–4). The results of these experiments show that the Mls bind the core4 sequence with comparable affinities with respect to the other core sequences tested and this demonstrates that the presence of A-tracts flanking the 5′-AAATAA3′ core does not appreciably alter the DNA-binding affinities of the Mls. As expected, the mutated version of core4 (core4mut) in which the central thymine is mutated to adenine (Fig. 4), does not bind to the Mls in EMSA experiments (Fig. 4, b, lane 4 and d, lane 4) even when a four-fold molar excess of proteins was added (Supplementary Fig. S4).

To further confirm the pivotal role of the T-A step, we designed a 20 bp oligonucleotide containing the sequence 5′-ATATAT-3′ (core 5; Fig. 4) for testing by EMSA (Fig. 4, b, lane 5 and d, lane 5) and in competion assays (Fig. 5, h, lanes 4–7 and l, lane 4–7). The core5 oligonucleotide is bound by Mls with comparable affinity in comparison to the other core oligonucleotides tested. Interestingly, the 5′-ATATAT-3′ sequence was found by Protein Binding Microarray (PBM) in several of the highest Z-score target sites of H-NS^17^, which also binds AT-rich DNA sequences containing T-A steps^17,25^. Taken together, these results demonstrate that the Mls are unable to bind A-tracts under the condition tested and an AT-rich sequence of five base pairs containing a central T-A step constitutes a core DNA-target site for Mls.

Many prokaryotic proteins, which bind AT-rich sequences in DNA make contacts with the minor groove and this includes the prokaryotic histone like proteins such as H-NS, TATA-box binding proteins and IHF (integration host factor)^16^. To test the hypothesis that the Mls also make contacts with DNA in the minor groove, we performed competition assays with the core2 (Fig. 5 a and c) and core5 (Fig. 5 b and d) sequences in the presence of netropsin. Netropsin is a naturally occurring polypyrrolecarboxamide that binds to the minor groove of AT-rich DNA and competes with H-NS for binding to DNA^17^. The results obtained show that Mls binding to core2 and core5 is significantly reduced when netropsin was added at a ratio of 1:1 with respect to DNA and this supports the idea that the Mls make important contact(s) with the minor groove when binding to DNA.

### MucR from *Brucella abortus* is able to recognize the Mls DNA-target site

**Figure 6:**
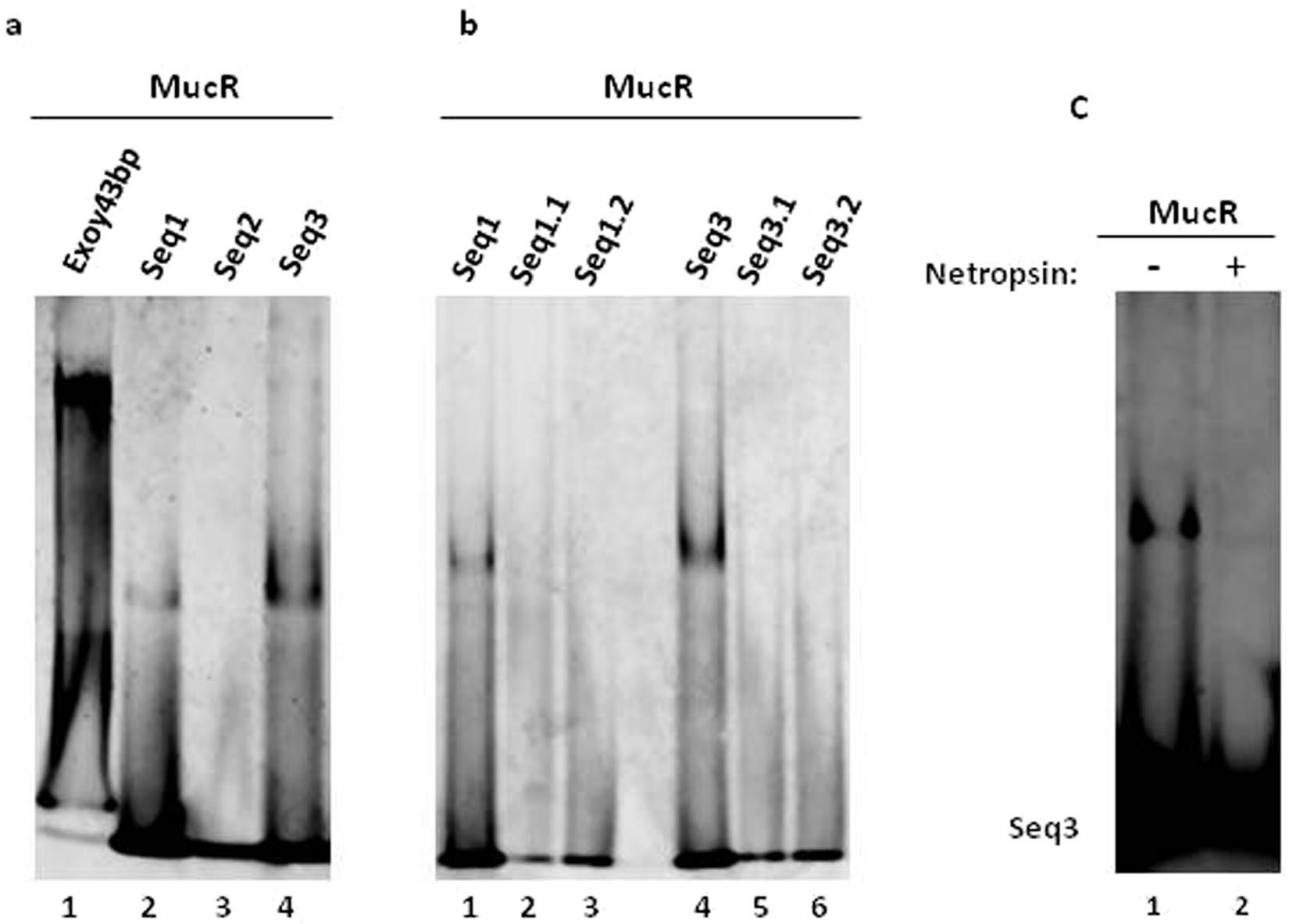
*Brucella* MucR binding to the target site for the Mls. (**a**) EMSA of MucR binding to the double-stranded oligonucleotides Exoy43bp, Seq1, Seq2 and Seq3; (**b**) EMSA of MucR binding to the double-stranded oligonucleotides Seq1.1, Seq1.2, Seq3.1 and Seq3.2; (**c**) competition assay of MucR binding to double-stranded Seq3 in the presence of a 1:1 molar ratio of netropsin. Full-length gels are presented in Supplementary Figs S31–S33.

The two Ml proteins studied both share considerable amino acid sequence identity with MucR and the amino acid sequence identity of Ml2 to MucR is 69% (Supplementary Fig. S5). In addition, it has been previously reported that the *Caulobacter crescentus* MucR recognizes degenerate AT-rich regions^6^. Given the high sequence identity between MucR and Ml2 and the ability of MucR to bind AT-rich regions, we investigated the ability of MucR to bind to various sequences in Exoy43bp. In EMSA experiments, the MucR from *B*. *abortus* is able to bind to the Exoy43bp, Seq1 and Seq3 oligonucleotides but fails to bind the Seq2 oligonucleotides, under the condition tested (Fig. 6 a). Consistent with their high sequence identity, these results are similar to what was observed with the Mls for the same oligonucleotides. To further demonstrate that MucR recognizes the same AT-rich sequence bound by Mls, we performed additional EMSA experiments with the mutated versions of Seq1 (Seq1.1 and Seq1.2) and Seq3 (Seq3.1 and Seq3.2) in which the core 5′-AAATA-3′ sequence is disrupted. As expected, the MucR does not bind the mutant oligonucleotides in the conditions tested (Fig. 6 b) and this indicates that it recognizes the same five base pair 5′-AAATA-3′ sequence as the Mls. In addition, we performed competition assays in the presence of netropsin at a ratio of 1:1 with respect to the Seq3 (Fig. 6 c), core2 and core5 sequences (Supplementary Fig. S6) and as expected MucR binding to these sequences is significantly reduced, which suggests that MucR and the Mls use similar mechanisms for DNA recognition.

### Mls proteins are able to oligomerize through the N-terminal region

It was previously reported that the MucR1 and MucR2 proteins from *Caulobacter crescentus* were able to form heterodimers^6^. Considering the high level of amino acid sequence identity between the Mls and MucR, we investigated whether or not Ml1 and Ml2 had the capacity to form oligomeric structures. By Dynamic Light Scattering (DLS) analysis, we found that the Mls hydrodynamic radius values were both around 6 nm (Table 1). These value are much higher than what would be expected if these proteins were in their monomeric state. To more throroughly investigate the formation of a quaternary structure, we performed a static Light Scattering (LS) analysis of all the protein samples. Interestingly, the data indicated that the molecular weight of the Mls is about 165 kDa, which is in perfect agreement with a decameric oligomer for Mls under the experimental conditions tested (Table 1). Next, we analyzed the oligomerization state of the region corresponding to just the DNA-binding domain of the Mls^1^. Under the condition tested, the radius measurement obtained by LS reveals a value around 2 nm and the molecular weights were found to be consistent with the monomeric state of the proteins (Table 1). The absence of any oligomeric form when only the C-terminal DNA binding domains were analyzed under the condition tested, suggest that the formation of higher-order oligomers with the full-length proteins is mediated by the N-terminal region. Given its high sequence identity to Ml1 and Ml2, we also investigated the oligomerization state of MucR from *B*. *abortus* and we found that also MucR forms higher-order oligomers under the conditions analyzed by LS. In fact, the molecular weight was determined to be 170 kDa (as reported in Table 1) and this also correspond to a decamer structure for MucR. In addition, its R_H_ value of about 6 nm is also consistent with what is observed for the Mls in this study.

**Table 1:** LS and DLS analysis of Mls and MucR; LS analysis of the region corresponding to the DNA-binding domain of the Mls.

Protein	Theoretical Molecular weight monomer	Experimental Molecular weight by Static LS	ExMw/ThMw	R_H_ by DLS
Ml1	16052 Da	168000 (±1,0%) Da	10.40	7.1 ± 11 nm
Ml2	15662 Da	156000 (±2,0%) Da	9.96	5.5 ± 0.4 nm
MucR	16024 Da	167000 (±1,0%) Da	10.40	6.3 ± 1.5 nm
Ml1_53–149_	10835	11160 Da	1.03	
Ml2_58–141_	9900	13410 Da	1.35	

### *ml2* fails to restore the wild type phenotype of the *Brucella* abortus CC092 mucR mutant strain

**Figure 7:**
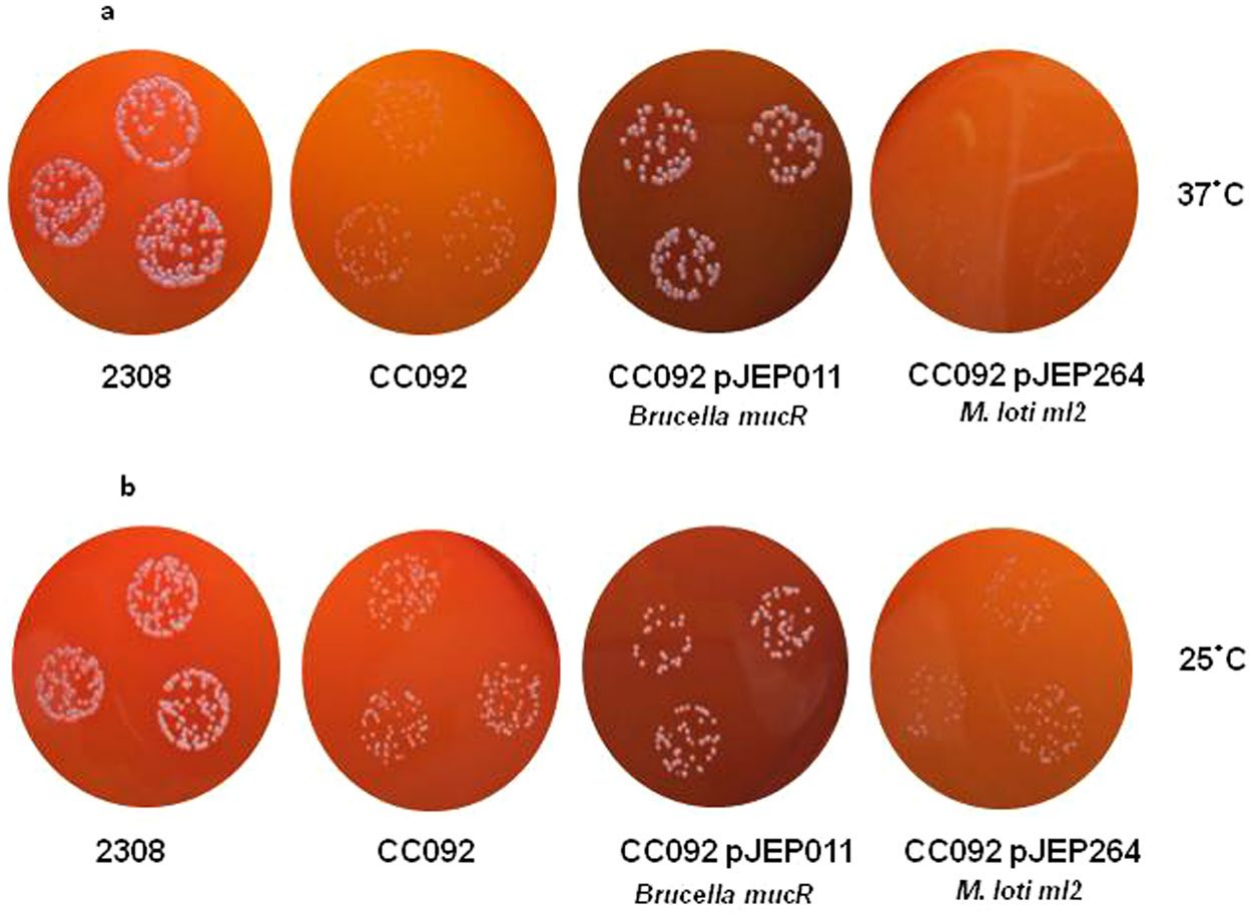
Colony formation by *B*. *abortus* 2308, an isogenic *mucR* mutant CC092, CC092 carrying a plasmid-borne copy of the *Brucella mucR* [CC092 (pJEP011) and CC092 carrying a plasmid-borne copy of the *M*. *loti ml2* under control of the *Brucella mucR* promoter [CC092 (pJEP264)] following 72 h incubation at 37 °C (**a**) and 168 h incubation at 25 °C (**b**) on Schaedler agar supplemented with 5% defibrinated bovine blood. Colony size of the *mucR* mutant CC092 is restored by transforming the mutant strain with pJEP011 containing the *mucR* gene, and its native promoter, whereas colony size is not restore by plasmid pJEP264 containing the *ml2* gene under control of *mucR* promoter, under the condition tested.

Based on the significant level of amino acid identity shared by the Ml proteins and the *Brucella* MucR, we investigated whether or not the Ml proteins had the capacity to rescue the distinctive growth defect phenotype present with the *B*. *abortus mucR* mutant CC092^2^. For this complementation experiment, we chose Ml2 because it has the highest amino acid sequence identity with the *Brucella* MucR. B. abortus *mucR* mutant CC092 was transformed by electroporation with the plasmid pJEP264 containing the *ml2* gene under the control of *mucR* promoter and the expression of *ml2* was verified by q-RT PCRs (Supplementary Fig. S7). In the complementation experiment, *ml2* is not able to restore the wild-type phenotype in the *B*. *abortus mucR* mutant under the conditions tested (Fig. 7a,b). These results suggest that although Mls proteins and MucR share considerable amino acid sequence identity and recognize the same DNA-target site, the wild-type regulatory activity of the *Brucella* MucR requires additional interactions with other cellular proteins and/or DNA that the Ml2 is not able to carry out.

## Discussion

*Rhizobium* spp. and *Brucella* spp. are members of the α2 subclass of proteobacteria, which includes bacteria that are symbionts and pathogens of plants and mammalian pathogens^2^. Due to the close phylogenetic relatedness of the α2-proteobacteria, these organisms use common genes and strategies for facilitating their interactions with their specific host, and the gene encoding the transcriptional regulator Ros/MucR is one of the genes conserved in the α2-proteobacteria that is important for host-bacterium interactions^2^. In this study, we show that four of the five *ml* genes belonging to Ros/MucR family, *ml1*, *ml2*, *ml3* and *ml5* are well expressed in *M*. *loti* during the stationary phase of planktonic growth whereas their expression level is very low during exponential phase. As in the case of MucR from *B*. *melitensis* ^26^, the Ml proteins seem to be necessary in stress conditions. In fact, during stationary phase when nutrients start to be limiting and in the presence of a high density of cell population, quorum sensing is activated to finely tune the expression of genes in response to different environmental conditions^27^. MucR in *S*. *meliloti* acts together with the quorum sensing cascade to regulate the expression of virulence genes as well as the enzymes necessary to produce exopolysaccharides^5^. Our results showing that *ml* genes are expressed in biofilms leads us to speculate that Ml proteins are involved in the regulation of exopolysaccharides biosynthesis, which are the major component, after water, of the extracellular matrix^19^. Together with the expression profile, the position of the new DNA-binding site identified for the Mls strongly suggests that Ml proteins are involved in regulating exopolysaccharide biosynthesis. In fact, the new target sequence is located in the region corresponding to the promoter of the *exoY* gene in *M*. *loti*, which indicates that the Ml proteins are transcription factors able to interact with the regulatory region of the *exoY* gene encoding a galactosyl transferase necessary for succinoglycan synthesis. One of the five *ml* gene studied, *ml4*, is not expressed in any condition tested. Nevertheless, the redundancy of the *ml* genes in *M*. *loti* leads us to speculate that there is an evolutionary pressure to assure the Ml proteins function. From this point of view, it could be that in the complex soil matrix, some particular environmental conditions, which cannot be replicated when culturing *M*. *loti* in TYR broth, activate *ml4* gene for a particular biological function.

In these studies, we show for the first time that the core DNA-target site recognized by Mls is a five base pair AT-rich sequence containing a T-A step and that these proteins make crucial contacts in the minor groove when they bind to DNA. Furthermore, we demonstrate that one of the most thoroughly characterized members of the Ros/MucR protein family, MucR from *B*. *abortus*, is also able to recognize the same target site as Mls and it utilizes a similar recognition mechanism that involves contacting the minor groove of DNA. The MucR DNA-binding data reported in this study are consistent with results previously published by Viollier P.H and coworkers^6^ demonstrating that MucR is able to recognize an AT-rich sequence in genomic GC-rich regions. In their study, a long degenerated DNA-target site for MucR from *C*. *crescentus* was identified. In this study, we clarify that a five base pair AT-rich sequence containing a T-A step is sufficient for the DNA-binding of MucR. The five base pair AT-rich sequence 5′-AAATA-3′ is also present in the VirC oligonucleotide, corresponding to the Vir Box, recognized by Ros from *A*. *tumefaciens* ^7^ and this suggests that the protein Ros also binds DNA using a similar mechanism as the Mls and MucR. The AT-rich sequences containing T-A step are preferential binding sites for many proteins that utilize a shape readout DNA recognition mechanism^16^. AT-rich sequences have a lower persistence length (the quantitative term for the stiffeness of a polymer) and they are more bendable than GC-rich sequences^16^. However, repetitive runs of four or more consecutive adenines (A-tracts) increases the rigidity of DNA strand and narrow the minor groove^16,28,29^. The T-A step is a flexible element of DNA that can serve to interrupt A-tracts and widen the minor groove^24,30^. For example, it plays a pivotal role in DNA recognition by TATA-Box binding proteins (TBP), which contact only the minor groove without base specific recognition^16^. A central T-A step was found in H-NS target sites^25^ and the pivotal role of this element in H-NS DNA recognition is investigated in detail demonstrating that many of the less-preferred 100%-AT 8-mers bound by H-NS contain A-tracts whereas AT-rich sequences containing T-A steps are present in several of the highest Z-score 8-mers identified by protein binding microarrays^17^. The preference of H-NS for AT-rich sequences containing T-A steps is explained by the geometry of the minor groove of DNA, which becomes optimal for H-NS binding when the target sequence contains T-A steps^17^. The results presented in this study suggest that the members of Ros/MucR protein family utilize a very similar DNA recognition mechanism to that of H-NS. H-NS contacts the DNA minor groove using an AT-hook-like loop^17,31^, which is located in the sequence “QGRTPA”^17^. Site-direct mutagenesis studies helped to demonstrate that the the QGR sequence was essential for H-NS DNA-binding and the most drastic reduction in DNA-binding was observed when the arginine residue was substitute by alanine^17^. Positively charged residues, such as arginine and at a less extent lysine, are the residues most frequently involved in making contacts with the minor groove of DNA when it is narrowed in AT-rich sequences^24^. The Ml proteins, MucR and Ros all share a conserved C-terminal basic arm necessary for interaction with DNA^1,7,8,32^. We speculate that the minor groove recognition occurs through positively charged amino acids in the C-terminal basic arm which are similar to sequences present in the AT-hook-like loop of H-NS. The role of the C-terminal basic arms of Ml proteins in DNA-binding is also supported by the model of the structure of Ros C-terminal DNA-binding domain with DNA^32^, which demonstrates that the basic arm is wrapped around DNA.

The C-terminal DNA-binding domains of the Ml proteins and the Ros protein have been well characterized^1,7,8,32^. Conversely, no data are available about the function of the N-terminal region of Ros/MucR family members. In this study, we show that the Ml1 and Ml2 proteins are able to form higher-order oligomers and that the region responsible for oligomerization is located in the N-terminal region of the proteins. Moreover, the results reported in this study show that the ability to form higher-order oligomers is also shared by MucR from *B*. *abortus*. It was previously reported that MucR1 and MucR2 from *Caulobacter crescentus* were able to form heterodimers^6^. Our data is the first evidence that the Ros/MucR family members have the capacity to form higher-order oligomers. However, we cannot exclude that under different experimental conditions the Mls and MucR could form lower-order oligomers such as dimers. As in the DNA recognition mechanism, the oligomerization by members of Ros/MucR protein family is similar to what is observed with the H-NS protein. H-NS binds high affinity nucleation sites through its C-terminal DNA-binding domain and it forms higher-order oligomers through their N-terminal region, which enables it to extend along the nucleoid^33,34^. Analyzing the DNA regions known to be bound by members of Ros/MucR protein family, AT-rich sequences containing T-A steps are generally separated by less than ten nucleotides. We speculate that the Ros/MucR protein family members behave as H-NS by first recognizing the AT-rich sequences through interactions with the C-terminal DNA-binding domain and then extending their presence along the nucleoid by oligomerizing through their N-terminal region. However, additional studies using longer DNA-target sites containing multiple nucleation sites are needed to better clarify the role of oligomerization in the DNA-binding of the Ml proteins and MucR. Finally, we demonstrate that even though Ml2 shares high sequence identity with MucR as well as the ability to recognize the same DNA sequence, it is not able to restore the wild type growth phenotype in the *B*. *abortus mucR* mutant strain. These data lead us to speculate that in prokaryotic cells the Ros/MucR family members participate in interactions with other protein partners and that these co-factors dictate the specific role of each member of the family. Altogether, the data present in our study show that four of the five *ml* genes studied are expressed in *M*. *loti* under planktonic condition as well as in biofilms and demonstrate that Ml proteins and MucR are able to recognize an AT-rich sequence of five base pairs containing a T-A step and oligomerize through the N-terminal region.

## Methods

### *M. loti* growth conditions and biofilm formation

Planktonic growth condition: *M*. *loti* were grown at 28 °C with vigorous shaking in TY broth supplemented with 5 mM CaCl_2_ (TYR). The exponential phase was reached at OD_600nm_ = 0.6, whereas the stationary phase was reached at OD_600nm_ = 2.

#### Biofilm formation

The biofilm formation was performed as previously reported^35^ with minor modifications. Briefly, an exponential phase culture of *M*. *loti* was diluted in fresh medium to OD_600_ = 0.1. Three ml of the diluted cell suspension was inoculated into multi-well cell culture plates. Wells containing only growth medium were used as negative controls. Plates were incubated at 28 °C for 24, 48, 72, 96 h under static conditions. At each time point, media and unattached cells were removed, the wells were rinsed three times with PBS and stained with 0.01% crystal violet solution for 20 min. Then, the crystal violet solution was removed and wells were rinsed three times with ultrapure water (Millipore). The crystal violet bound to wells was solubilised with 95% ethanol. The same treatment with crystal violet solution was perfomed on empty wells as a control. Biofilm formation was quantified by measuring the OD_595nm_ for each well using a microplate reader (Synergy HT Biotek).

### RNA extraction and Real Time PCR

Total RNA was extracted from *M*. *loti* planktonic cells grown to either exponential phase or stationary phase by RNeasy Mini Kit (Qiagen) and from *M*. *loti* biofilms formed after 24 h or 48 h by PowerBiofilm RNA Isolation Kit (Mo Bio). The extracted RNA was quantified using a NanoDrop 2000c spectrometer (ThermoScientific), purity was controlled by A_260_/A_280_ and A_260_/A_230_ ratios and integrity by electrophoresis on 1.5% agarose gel. Contaminating chromosomal DNA was removed by digestion with RNase-free RQ1 DNase (1U/μl; Promega) for 15 min at 37 °C followed by RNA precipitation with 0.3 M Na-acetate and two volumes of 95% ethanol. The pellet was resuspended in RNase-free ultrapure water (Millipore).

One μg of total RNA from *M*. *loti* planktonic cells or from biofilms was reverse transcribed to cDNA by 5X All-In-One RT MasterMix (with AccuRT Genomic DNA Removal Kit - abm). Real time PCRs was performed using 12.5 μl of 2x Sybr green master mix (Applied Biosystems); 12.5 pmol of each primer (sequences reported in Supplementary Table S1) and 5 μl of cDNA in a total volume of 25 μl. For each primer set, RT negative samples were performed as control. The reactions were performed using the following program: 95 °C for 10 min; (95 °C for 15 sec; 60 °C for 1 min; 72 °C for 1 min) repeated for 40 cycles. A dissociation stage was added at the end of each run to confirm the amplification of a specific transcript. Three housekeeping genes, *rpoA*, *rpoD* and *16S* were used. The primers were designed by the program Primer3 Plus on the basis of the gene coding sequences to obtain amplicons of approximately 160 bp. The results for each condition were confirmed by two biological and three technical replications. Data were analysed by the 2^−ΔΔCt^ method. Student T-test was used to evaluate the statistical significance of the results.

### Cloning, protein expression and purification

The *ml* genes were amplified by PCR from *M*. *loti* genomic DNA extracted using ULTRAprep Genomic DNA kit (miniprep genomic DNA BAC – Top line). The oligonucleotides used as primers were designed on the basis of the *M*. *loti* genome sequence^20^. The *Brucella mucR* was amplified by PCR from the plasmid pJEP101. This plasmid was originally constructed by amplifying the *mucR* gene (BAB1_0594) and its native promoter from genomic DNA from *B*. *abortus* 2308 by PCR using primers mucRpMR10HindF (gccaagcttctcaattttcttgcggtgccctg) and mucRpMR10EcoR1R (agtgaattctcaggcgtccttcggcttgcgg) and the Midas PCR Mix (Monserate). The resulting DNA fragment was ligated into pGemTEasy (Promega) by TA cloning. The *mucR* gene from *B*. *abortus* was, then, amplified by PCR from the pGEM T easy kit containing the *mucR* gene and its promoter sequence^2^. The sequences of the primers used are reported in Supplementary Table S1. All PCR products were digested with NdeI and EcoRI restriction enzymes (New England Biolabs) and cloned into NdeI/EcoRI digested pET-22b(+). The plasmid clones used to express the *ml* gene regions encoding the DNA-binding domains of the Ml proteins were previously reported^1^. The Ml1 and Ml2 full-lenght were expressed in *E*. *coli* host strain BL21(DE3), induced for 1 h at 28 °C with 1 mM IPTG. The Ml protein fragments containing the DNA-binding domains were expressed in *E*. *coli* host strain BL21(DE3) and induced for 2 h at 37 °C. For protein purification, we used the previously reported method^1^. Ml1, Ml2 and MucR were eluted from a Mono S HR 5/5 cation exchange chromatography column in the 0.4–0.8 M NaCl concentration range.

### Electrophoretic Mobility Shift Assay (EMSA)

The EMSA experiments were performed as previously described^36^. Briefly, 0.5 μg of each protein was incubated 10 min on ice with 2.5 pmol of double-stranded oligonucleotides (sequences reported in Supplementary Table S2) in binding buffer and then loaded onto a 5% polyacrylamide gel in 0.5X TBE (200 V for 70 min). A two- or four-fold excess of proteins was used, as indicated in each figure. The competition experiments were conducted as previously reported^37^. Briefly, 0.5 μg of protein were incubated ten minutes on ice with the FAM-labelled double stranded Seq3 oligonucleotide (sequence reported in Supplementary Table S2) in binding buffer. Then, an excess of unlabelled competitor DNA (amounts indicated in each figure) was added to the reaction mixture. After 10 min of incubation on ice with the competitor, the samples were loaded onto a 5% polyacrylamide gel in 0.5X TBE (200 V for 70 min). In the competition assays with netropsin, a netropsin:DNA ratio of 1:1 was used. The polyacrylamide gels were imaged on a Typhoon Trio + scanner (GE Healthcare).

### Dynamic Light Scattering (DLS) and Light Scattering

Size measurements of Ml1, Ml2 and MucR were performed on a Nano Zetasizer, spectrometer (Malvern, UK) at concentration 1 mg/ml and of 0.5 mg/ml with the same buffer conditions used for the final purification procedures^38^. The wavelength of the laser was set at 632.8 nm and the scattering angle was 173°. For each sample, the mean value of the particle diameter was calculated from three replicate determinations. The molecular diameter is calculated from the autocorrelation function of the intensity of light scattered from the particles assuming a spherical particle form. For molecular weight measurements, a MiniDAWN Treos spectrometer (Wyatt Instrument Technology Corp.) equipped with a laser operating at 658 nm was used connected on-line to a size-exclusion chromatography. Samples at 1 mg/ml were loaded onto a Superdex 200 column (10 × 30 cm, GEHealthcare) equilibrated in the same buffer used for the final purification procedure and connected to a triple-angle light scattering detector equipped with a QELS (Quasi-Elastic Light Scattering) module^39^. A constant flow rate of 0.5 ml/min was applied. Elution profiles were detected by a Shodex interferometric refractometer and a mini Dawn TREOS light scattering system. Data were analyzed using the Astra 5.3.4.14 software (Wyatt Technology).

### Genetic complementation of a *Brucella mucR* mutant

Genetic complementation of the *B*. *abortus mucR* mutant CC092^2^ with a wild-type version of the corresponding gene was performed by expressing this gene from its native promoter in pMR10^40^. The *mucR* gene (BAB1_0594 in the *B*. *abortus* 2308 genome sequence) and its native promoter were amplified by PCR using the primers reported in Supplementary Table S1, with the *Q5* polymerase (NEB) and *B*. *abortus* 2308 genomic DNA serving as templates. The resulting DNA fragment was digested with HindIII and EcoRI and ligated into HindIII/EcoRI digested pMR10. This plasmid, pJEP011, was introduced into the *B*. *abortus mucR* mutant strain CC092 by electroporation. Heterologous production of the *M*. *loti* Ml2 protein in *B*. *abortus* CC092 was performed by expressing the wild-type *ml2* allele from the *mucR* promoter in pMR10. A hybrid DNA sequence containing the *Brucella mucR* promoter region extending through the first codon of the *Brucella mucR* coding region fused to codons 2–141 of the *M*. *loti ml2* coding region was synthesized. This synthetic DNA fragment contains a native MfeI restriction site on the 5′ end and an engineered EcoRI site on the 3′ end. The synthesized DNA was digested with MfeI and EcoRI and ligated into MfeI/EcoRI digested pMR10, to form pJEP264. This plasmid was introduced into the *B*. *abortus* CC092 by electroporation. The expression levels of *ml2* gene and of *mucR gene* in *B*. *abortus mucR* mutant CC092 were verified by real time PCRs (primer sequences are reported in Supplementary Table S1). 16S gene was used as housekeeping gene (primer sequences for 16S real time are reported in Supplementary Table S1). The reactions were performed by the following program: 95 °C for 3 min; (95 °C for 15 sec; 55 °C for 30 sec; 72 °C for 30 sec) repeated for 40 cycles. A dissociation stage was added at the end of each run to confirm the amplification of a specific transcript. Bio-RAD iQ syber green 2x mix was used following the manufacturer’s protocol. Data were analysed by the 2^−ΔΔCt^ method. Student T-test was used to evaluate the statistical significance of results.

All experiments with live *Brucella* strains were conducted under Biosafety Level 3 conditions in compliance with regulations administered by the United States Center for Disease Control and Prevention’s Division of Select Agents and Toxins.

All data generated or analysed during this study are included in this published article (and its Supplementary Information files).

## Electronic supplementary material

Supplementary Information
