# Supplementary material for: Ml proteins from Mesorhizobium loti and MucR from Brucella abortus: an AT-rich core DNA-target site and oligomerization ability
Source: Sci Rep. 2017 Nov 17;7:15805. doi: 10.1038/s41598-017-16127-5 (PMC5693944; doi:10.1038/s41598-017-16127-5)
Supplement: Supplementary file 1 — Supplementary Information [file 41598_2017_16127_MOESM1_ESM.doc]

**Ml proteins from *Mesorhizobium loti* and MucR from *Brucella abortus*: an AT-rich core DNA-target site and oligomerization ability.**

Ilaria Baglivo^1*^, Luciano Pirone^2^, Emilia Maria Pedone^2^, Joshua Edison Pitzer^3^, Lidia Muscariello^1^, Maria Michela Marino^1^, Gaetano Malgieri^1^, Andrea Freschi^1^, Angela Chambery^1^, Roy-Martin Roop II^3^, Paolo Vincenzo Pedone^1*^.

^1^ Department of Environmental, Biological and Pharmaceutical Sciences and Technologies, University of Campania, Caserta, 81100, Italy

^2^ Institute of Biostructures and Bioimaging, C.N.R., Naples, 80134, Italy

^3^ Department of Microbiology and Immunology, Brody School of Medicine, East Carolina University, Greenville, NC, USA

**Supplementary Information.**

**Supplementary Table S1. Primer sequences for cloning and for real time experiments.**

| **gene** | **Primer forward (5’- 3’)** | **Primer reverse (5’- 3’)** |
| --- | --- | --- |
| *ml1* | CGGAATTCCATAATGACAGAAGAAGCAGAC | CGGAATTCGAACGAAGCTTGGGCGATAGTG |
| *ml2* | CAGCATATGGATATTGTCGAAACACC | CGGAATTCGCCAGCCGGCAATCCTGTTC |
| *ml5* | CAGCATATGACCGAAGAAACCGAGAGCAAAGCCG | CGGAATTCCCTATCCTGAAAAAGTGTGG |
| *mucR_pet22b+_* | CGGAATTCCATAATGGAAAATCTGGAAACG | CGGAATTCTCAGGCGTCCTTCGGCTTG |
| *mucR_pMR10_* | gccaagcttctcaattttcttgcggtgccctg | agtgaattctcaggcgtccttcggcttgcgg |
| *ml1_real time_* | GCAGACAAAAACATCGACACCCTC | CAGTGCTGCATGCACCTGGCC |
| *ml2_real time_* | ACACCTTCCAGAAACAACGATGCGC | CCACACGCCCGAGAGCGGC |
| *ml3_real time_* | CGCGGTCGTCGTGGAAAGCC | AACCATAGTCTGTCCTGAGGTGTC |
| *ml4_real time_* | GAAAGCCCTTGACCGACGAGAACA | CGTCCGACCTTCGATAAAGACAGG |
| *ml5_real time_* | CCGAGAGCAAAGCCGACAACC | CAATGCGATGTGGATCTGGCCG |
| *rpoA_real time_* | TGAACATCAAGGAGATCGCCA | AGCGTGCAGATGACGTGGTC |
| *16S_real time_* | TCCGAACTGAGATGGCTTTT | AGCATTCAGTTGGGCACTCT |
| *rpoD_real time_* | CCTGATCCAGGAAGGCAATA | TCGTCTCGATCATGTGAACC |
| *mucR_real time_* | CTGCTTTTGAGTTTGACCGC | GAACTTCAGCAATCAGAACCG |
|  |  |  |
|  |  |  |
|  |  |  |
|  |  |  |
|  |  |  |
|  |  |  |
|  |  |  |
|  |  |  |
|  |  |  |
|  |  |  |
|  |  |  |
|  |  |  |
|  |  |  |

**Supplementary Table S2. Oligonucleotide sequences used as target sites in EMSA experiments. The forward and reverse oligonucleotides were annealed and tested as double stranded**

| **Double-stranded oligonucleotide** | **Forward strand (5’-3’)** | **Reverse strand (5’-3’)** |
| --- | --- | --- |
| **Exoy43bp** | CCACTGAAATAGTCGCGTCATGAATTGACTAAATAATAAGCTA | TAGCTTATTATTTAGTCAATTCATGACGCGACTATTTCAGTGG |
| **Seq1** | CCACTGAAATAGTCGCGTCA | TGACGCGACTATTTCAGTGG |
| **Seq2** | TCGCGTCATGAATTGACTAA | TTAGTCAATTCATGACGCGA |
| **Seq3** | ATTGACTAAATAATAAGCTA | TAGCTTATTATTTAGTCAAT |
| **Seq1.1** | CCACGTCCCTAGTCGCGTCA | TGACGCGACTAGGGACGTGG |
| **Seq1.2** | CCACTGACCGCGTCGCGTCA | TGACGCGACGCGGTCAGTGG |
| **Seq3.1** | ATTGACGCCCTAATAAGCTA | TAGCTTATTAGGGCGTCAAT |
| **Seq3.2** | ATTGACTAAAGCCGAAGCTA | TAGCTTCGGCTTTAGTCAAT |
| **Core1** | GGCGCCGAAATAGCGGAGCG | CGCTCCGCTATTTCGGCGCC |
| **Core2** | GGCGCCGAAATAACGGAGCG | CGCTCCGTTATTTCGGCGCC |
| **Core2mut** | GGCGCCGAAAAAACGGAGCG | CGCTCCGTTTTTTCGGCGCC |
| **Core3** | GGCGCCGAAATACCGGAGCG | CGCTCCGGTATTTCGGCGCC |
| **Core4** | GCGAAAAAAATAATTTTGCC | GGCAAAATTATTTTTTTCGC |
| **Core4mut** | GCGAAAAAAAAAATTTTGCC | GGCAAAATTTTTTTTTTCGC |

**Supplementary Fig. S1.** Sequence alignment between the Vir Box from *A. tumefaciens* and Exoy43bp from *M. loti*. The sequence alignment was obtained by ClustalW (<http://www.genome.jp/tools/clustalw/>). A schematic representation of the position in the *M. loti* genome of the Exoy43bp sequence is also reported.

**
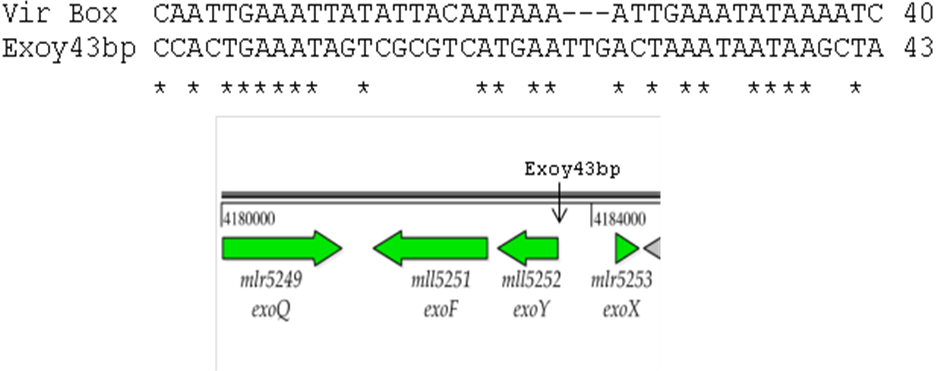
**

Exoy43bp

**Supplementary Fig. S2.** Ml proteins amino acid sequence alignment obtained by ClustalW (<http://www.genome.jp/tools/clustalw/>).

Ml1 --MTEEADKNIDTLIELTADVVSAYVSNNPVPVGDLPALIGQVHAALKGTAGFVSAAKPE 58

Ml5 --MTEETESKADNLIELTAHVVSAYVSNNPVPVGELPGLIGQIHIALKGTAGGAAPEKSE 58

Ml2 MDIVETPSRNNDALIELTADVVAAYVSNNPVPVGELPNLISDVHAALGRVGGTAEQPPAD 60

Ml3 --MKELSNIEDKTVIELTADIVSAYVGNNPLPASGLPDLIASVSASVRKLAGAVVVESP- 57

: * .. : . :*****.:*:***.***:*.. ** **..: :: .* . .

Ml1 ALEPAVPIRKSVTPDYIICLDDGKKFKSLKRHLSTHHGLTPDEYRAKWHLPADYPMVAPN 118

Ml5 ALKPAVPIRKSVTPDYIISLEDGKKFKSLKRHLATHYGLTPDEYRAKWELPADYPMVAPN 118

Ml2 KQKPAVNPKRSVHDDYIVCLEDGKKFKSLKRHLMTHYDLTPDQYREKWNLDPSYPMVAPN 120

Ml3 SLVPAVNPKKSVFPDYIICLEDGKKFKSLKRHLRTDYGLSPDDYRAKWGLPPDYPMVAPN 117

*** ::** ***:.*:************ *.:.*:**:** ** * ..*******

Ml1 YAAARSALAKTMGLGRKPKEPE-----ARTRKKAAA 149

Ml5 YAAARSALAKTMGLGRKPKEPETPAPAKRARKKAAA 154

Ml2 YAAARSQLAKKMGLGRK----------RKAR----- 141

Ml3 YSATRSALAKSTGLGRKPAAAP-AAVAKKGKAKA-- 150

*:*:** ***. ***** : :

**Supplementary Fig. S3.** EMSAs with growing amount of Ml1**(a)** and Ml2 **(b)** on the oligonucleotides Seq1, Seq2, Seq3. The amounts of protein used are 0.5 μg, 1 μg, 2 μg. The amount of DNA is constant for each lane.

**
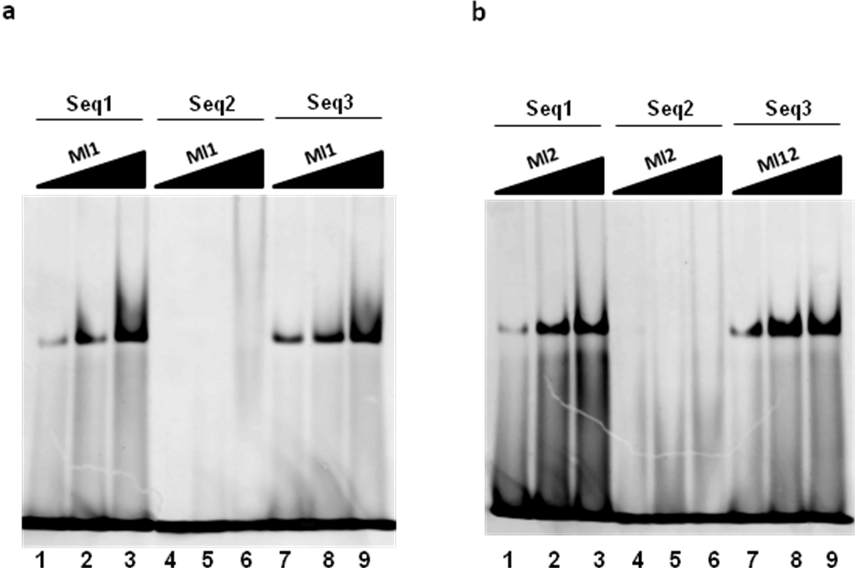
**

**Supplementary Fig. S4.** EMSAs with growing amount of Ml1(a) and Ml2(b) on the oligonucleotides core2mut and core4mut. The amounts of protein used are 0.5 μg, 1 μg, 2 μg. The amount of DNA is constant for each lane. As controls, EMSAs of Ml1 and Ml2 with core2 and core4 were performed as indicated in the Methods section.

**
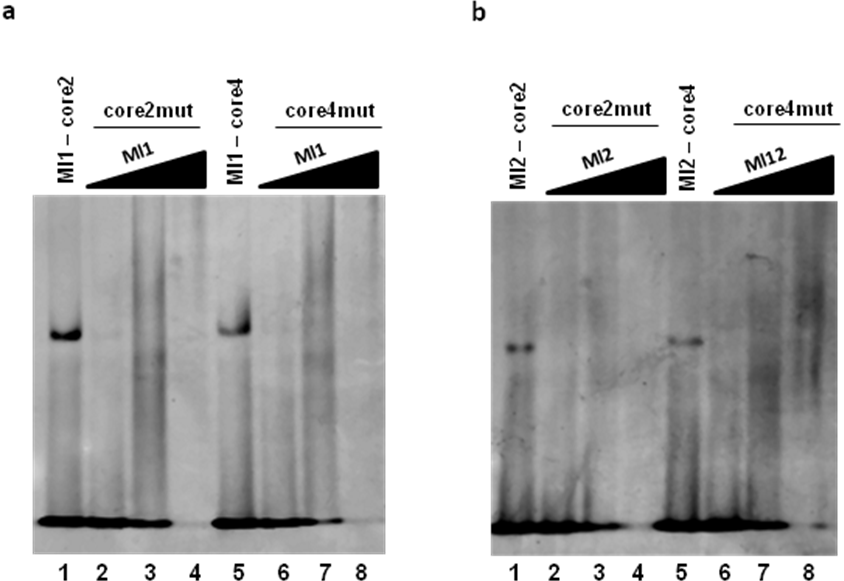
**

**Supplementary Fig. S5.** Ml2-MucR amino acid sequences alignment obtained by ClustalW (<http://www.genome.jp/tools/clustalw/>). The sequence identity calculated counting the conserved positions (asterisks) results to be 69%.

MucR_Babortus MENLETNDESTELLLSLTADVVAAYVGNNSIRAGELPVLIAEVHAAFKRH

Ml2_Mloti MDIVETPSRNNDALIELTADVVAAYVSNNPVPVGELPNLISDVHAALGRV

*: :** ....: *:.**********.**.: .**** **::****: *

MucR_Babortus VEREEAPVVVEKPKPAVNPKKSVHDDYIVCLEDGKKFKSLKRHLVTHYNM

Ml2_Mloti GGTAEQPPA-DKQKPAVNPKRSVHDDYIVCLEDGKKFKSLKRHLMTHYDL

* * . .* *******:***********************:***::

MucR_Babortus TPEQYREKWDLDPNYPMVAPNYAAARSRLAKKMGLGRKPKDA-

Ml2_Mloti TPDQYREKWNLDPSYPMVAPNYAAARSQLAKKMGLGRKRKAR-

**:******:***.*************:********** *

**Supplementary Fig. S6.** Competition assay of MucR binding to core2 and core5. The netropsin competes MucR DNA-binding at a ratio of 1:1 with respect to DNA

**
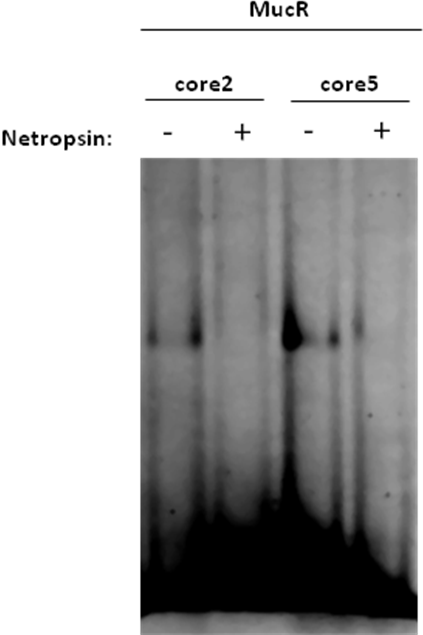
**

**Supplementary Fig. S7. a)** Relative expression levels of *mucR* in Brucella abortus wild-type strain 2308, in *Brucella abortus* *mucR* mutant strain CC092 trasfomed by pJEP011 plasmid containing *mucR*-promoter- *mucR* gene fragment and in in Brucella abortus *mucR* mutant strain CC092 transformed by pJEP264 containing *mucR*-promoter-*ml2* gene fragment; (**b**) relative expression levels of *ml2* in Brucella abortus wild-type strain 2308, in *Brucella abortus mucR* mutant strain CC092 trasfomed by pJEP011 plasmid containing *mucR*-promoter- *mucR* gene fragment and in *Brucella abortus mucR* mutant strain CC092 trasformed by pJEP264 plasmid containing *mucR*-promoter- *ml2* gene fragment transformed by pJEP264 containing mucR-promoter- *ml2* gene fragment.

The *ml2* gene is expressed in the mutant strain CC092 under the *mucR* promoter. For all the RT-qPCR values t-test, P<0,05.

**(a)**

**(b)**

**
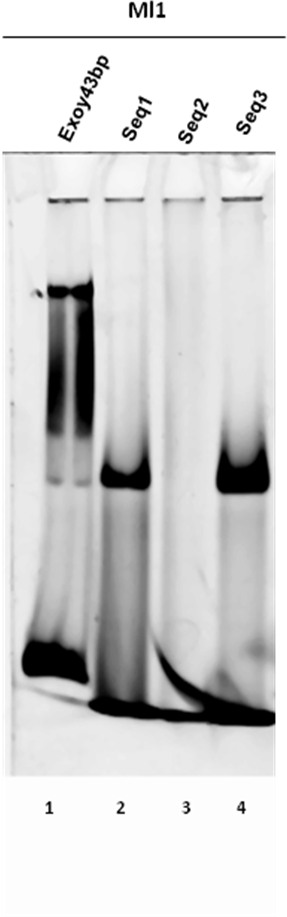
**

**Supplementary Fig. S8.** The full-length gel of the main figure 2 a.

**
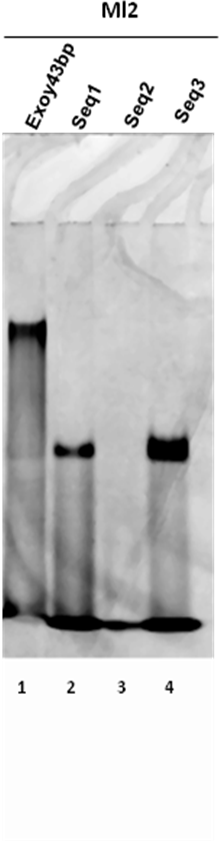
**

**Supplementary Fig. S9.** The full-length gel of the main figure 2 b.

**
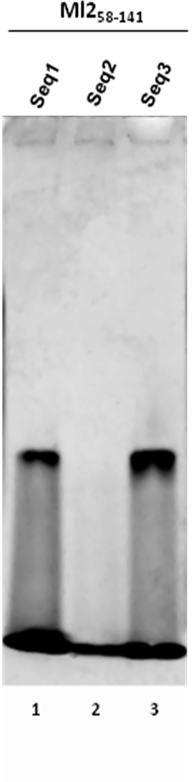
**

**Supplementary Fig. S10.** The full-length gel of the main figure 2 c.

**
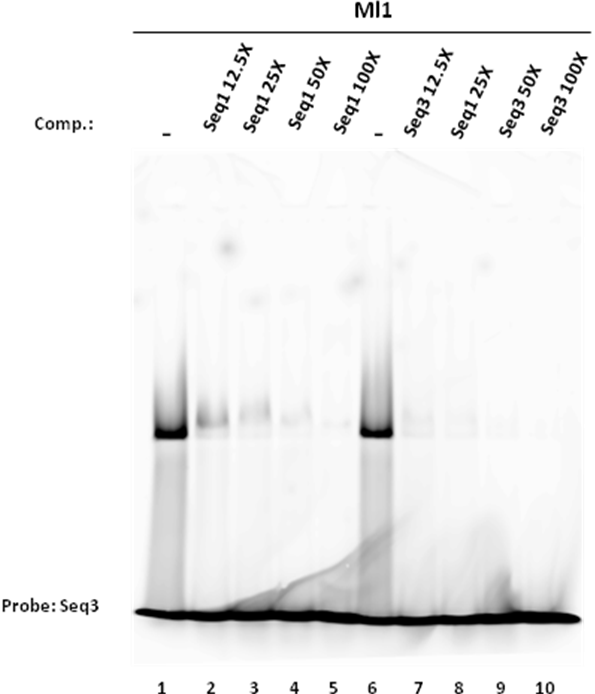
**

**Supplementary Fig. S11.** The full-length gel of the main figure 2 d.

**Supplementary Fig. S12.** The full-length gel of the main figure 2 e.

**
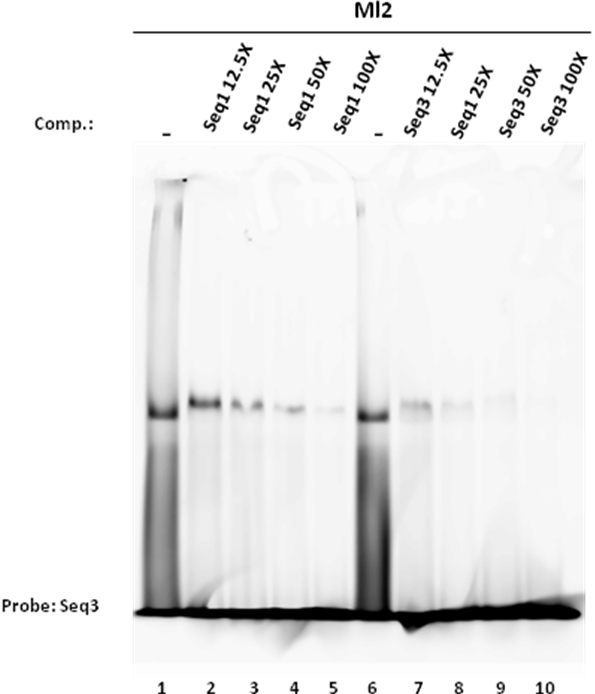
**

**
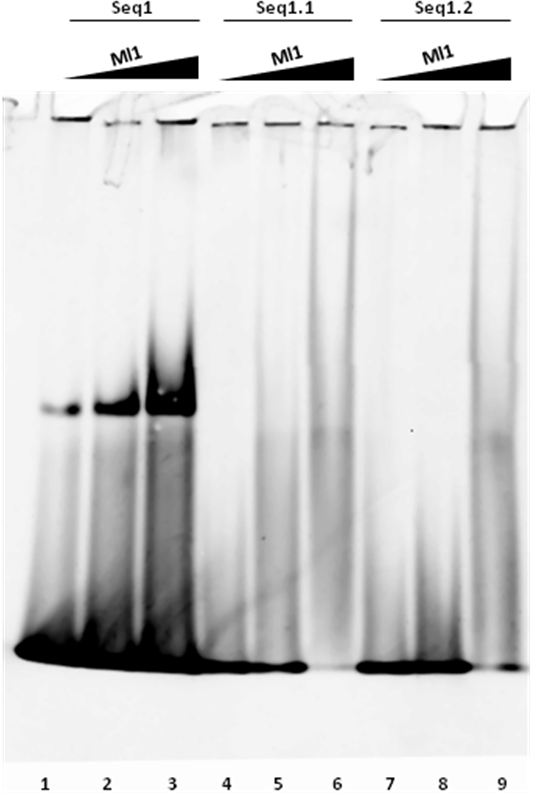
**

**Supplementary Fig. S13.** The full-length gel of the main figure 3 a.

**
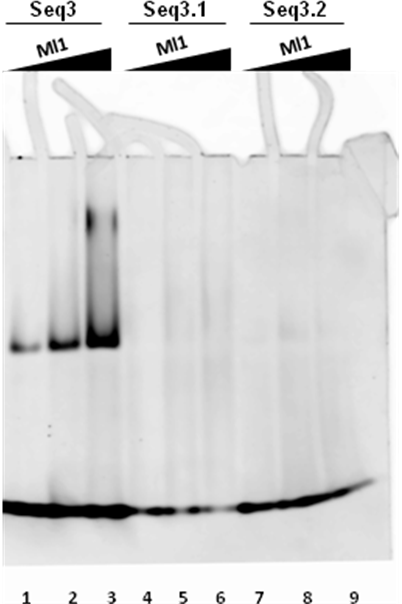
**

**Supplementary Fig. S14.** The full-length gel of the main figure 3 b.

**
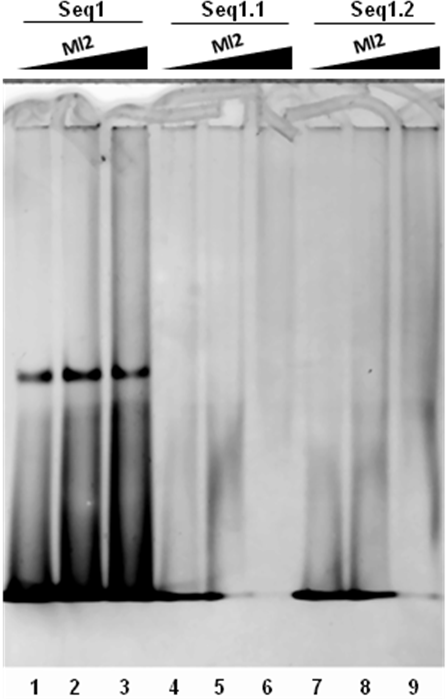
**

**Supplementary Fig. S15.** The full-length gel of the main figure 3 c.

**
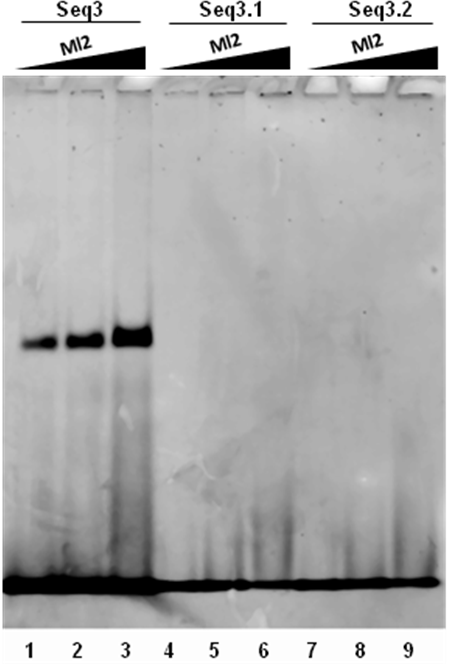
**

**Supplementary Fig. S16.** The full-length gel of the main figure 3 d.

**Supplementary Fig. S17.** The full-length gel of the main figure 4 a.

**
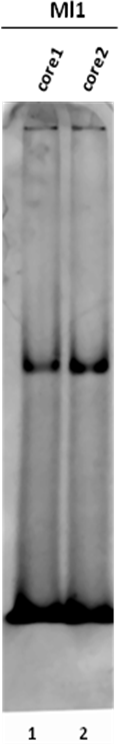
**

**
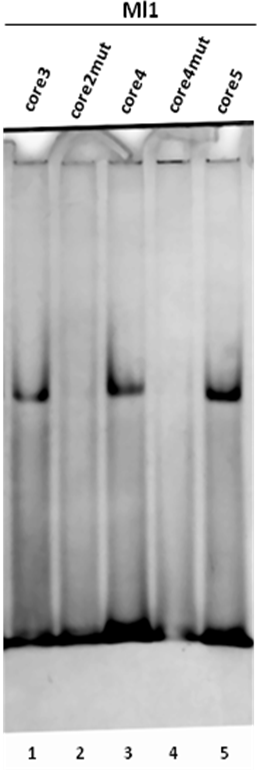
**

**Supplementary Fig. S18.** The full-length gel of the main figure 4 b.

**
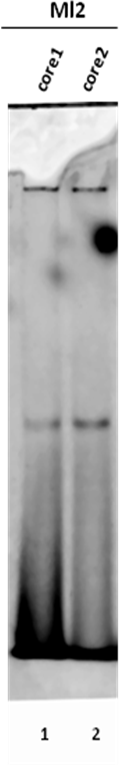
**

**Supplementary Fig. S19.** The full-length gel of the main figure 4 c.

**
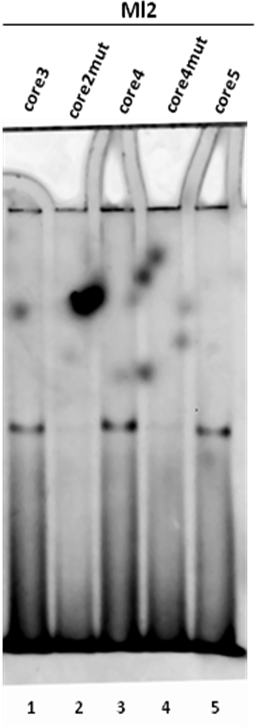
**

**Supplementary Fig. S20.** The full-length gel of the main figure 4 d.

**
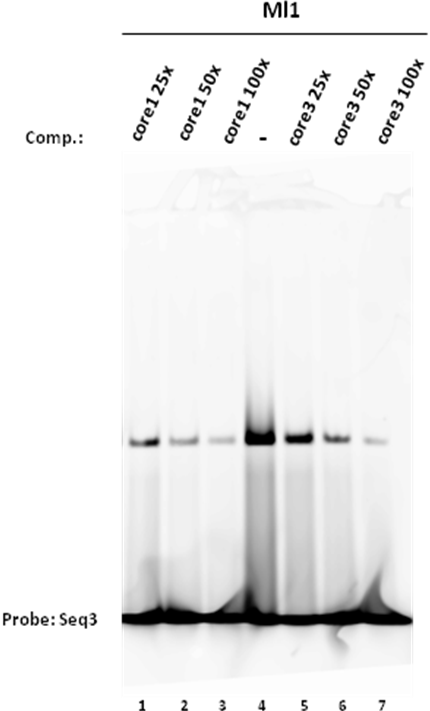
**

**Supplementary Fig. S21.** The full-length gel of the main figure 4 e.

**Supplementary Fig. S22.** The full-length gel of the main figure 4 f.

**
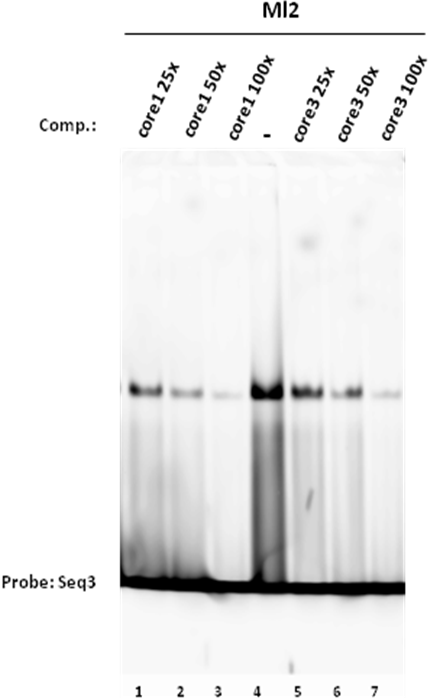
**

**Supplementary Fig. S23.** The full-length gel of the main figure 4 g.

**
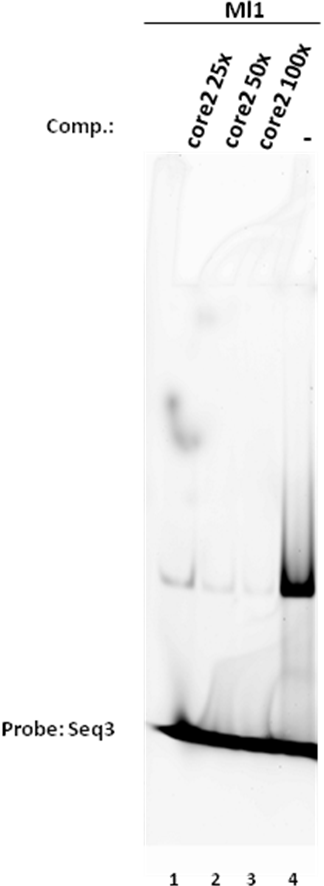
**

**Supplementary Fig. S24.** The full-length gel of the main figure 4 h.

**
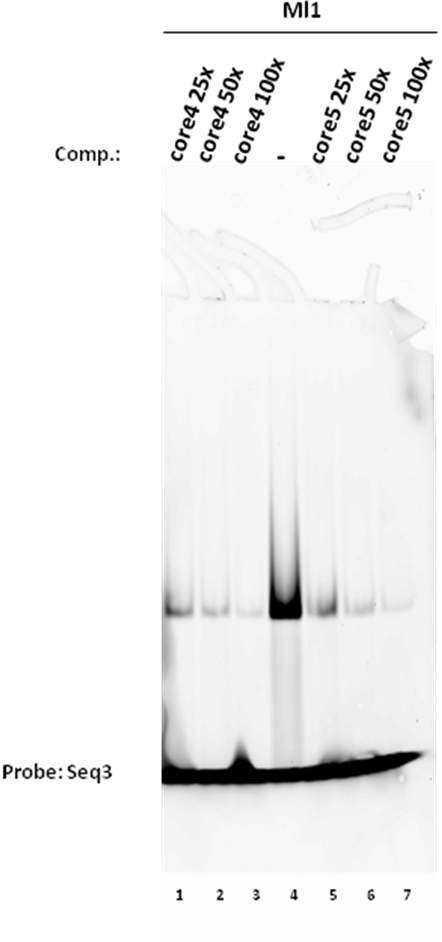
**

**Supplementary Fig. S25.** The full-length gel of the main figure 4 i.

**
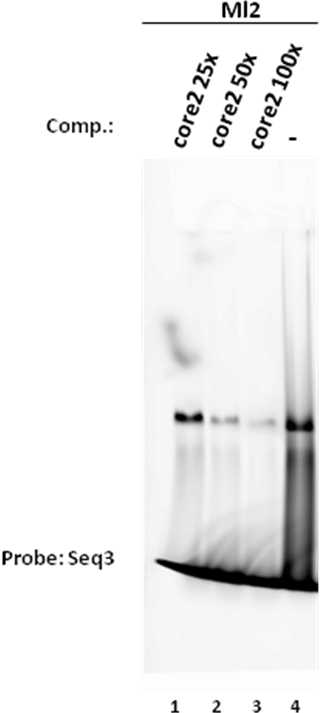
**

**Supplementary Fig. S26.** The full-length gel of the main figure 4 l.

**
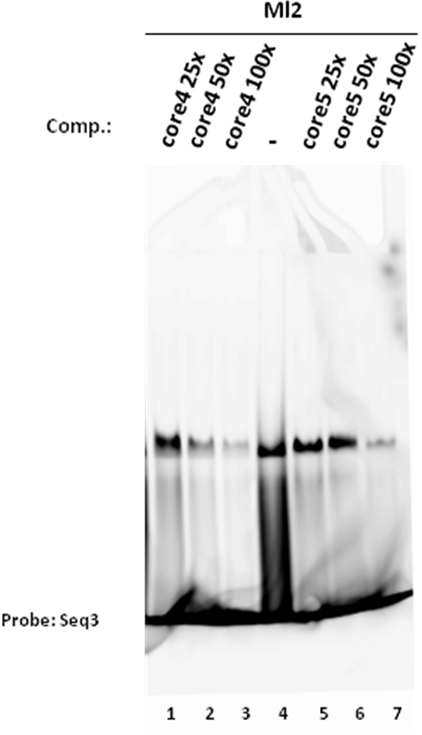
**

**
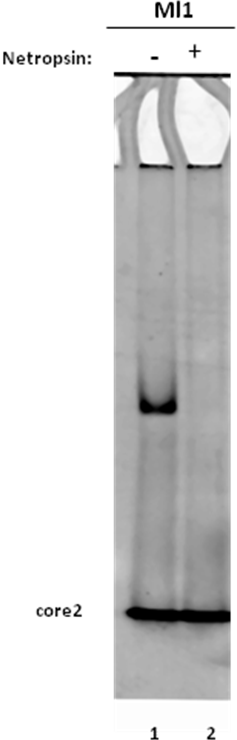
**

**Supplementary Fig. S27.** The full-length gel of the main figure 5 a.

**Supplementary Fig. S28.** The full-length gel of the main figure 5 b.

**
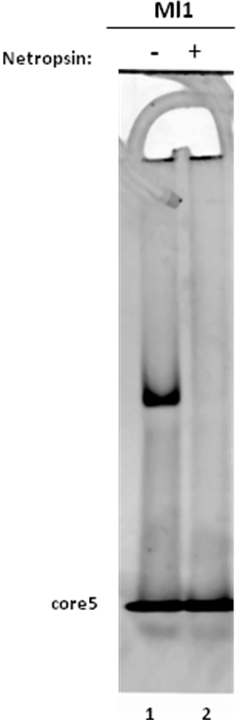
**

**Supplementary Fig. S29.** The full-length gel of the main figure 5 c.

**
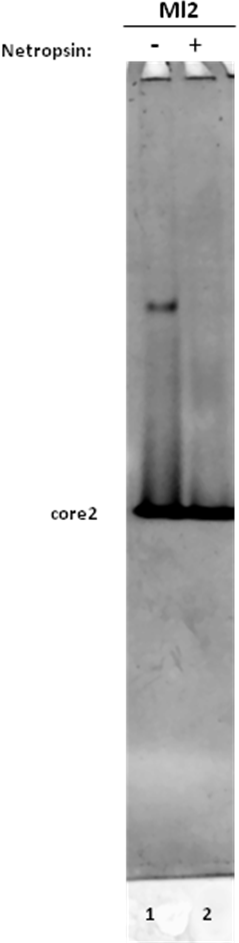
**

**Supplementary Fig. S30.** The full-length gel of the main figure 5 d.

**
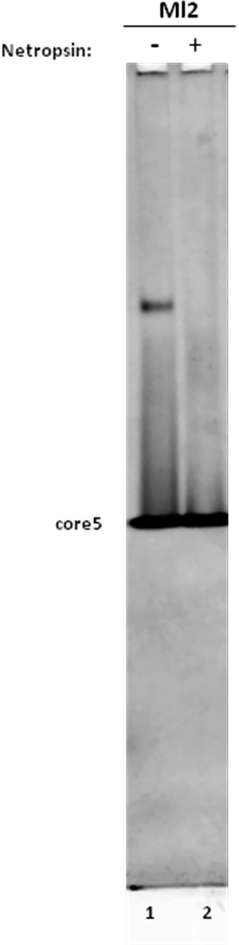
**

**
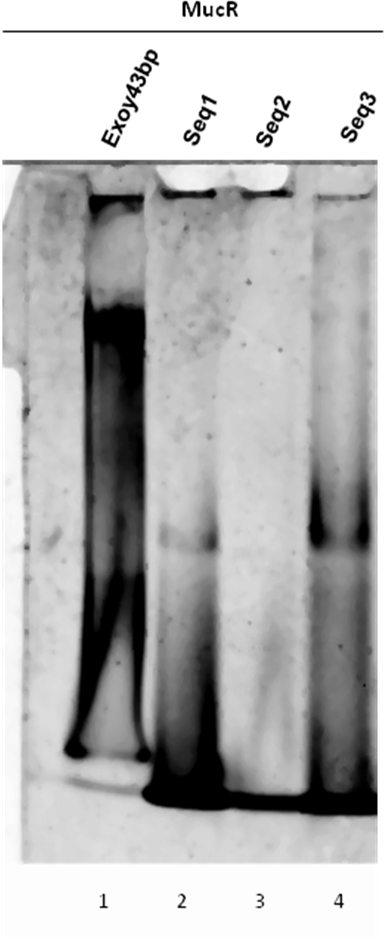
**

**Supplementary Fig. S31.** The full-length gel of the main figure 6 a.

**Supplementary Fig. S32.** The full-length gel of the main figure 6 b.

**
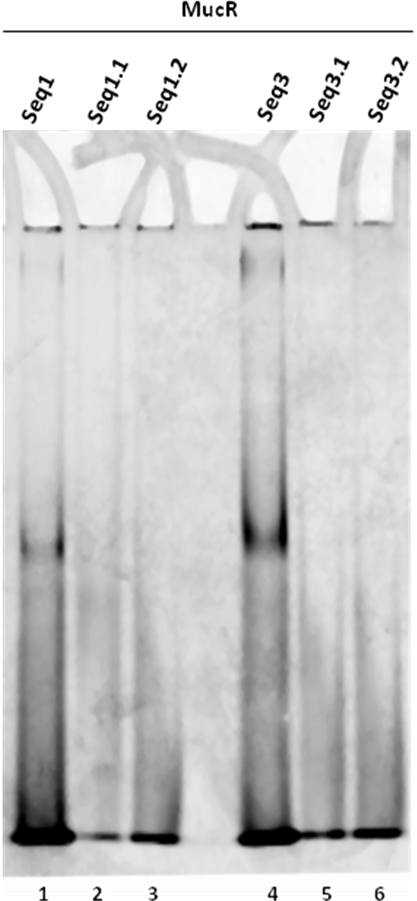
**

**
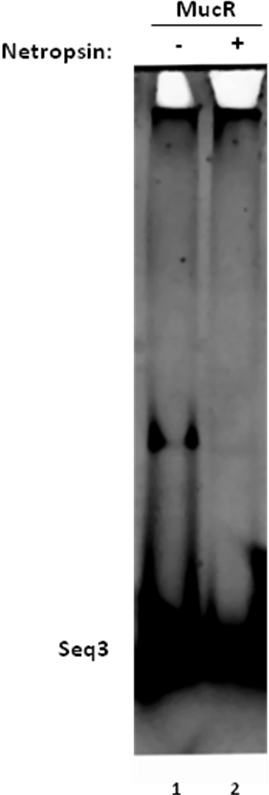
**

**Supplementary Fig. S33.** The full-length gel of the main figure 6 c.
